# Comprehensive 3-Year Pediatric Resident Resuscitation Simulation Curriculum on the Care of the Severely Ill Pediatric Patient

**DOI:** 10.15766/mep_2374-8265.11650

**Published:** 2026-11-06

**Authors:** Jessica Kent, Amanda Rogers

**Affiliations:** 1 Medical Student, Medical College of Wisconsin; 2 Associate Professor, Department of Pediatrics, Section of Hospital Medicine, Medical College of Wisconsin

**Keywords:** Simulation, Pediatrics, Codes, Acutely Ill, Shock, Arrhythmia, Respiratory Failure, Procedure Training, Hospital Medicine, Pediatric Critical Care Medicine, Pediatric Emergency Medicine

## Abstract

**Introduction:**

Pediatric residents must be able to manage severely ill patients by the end of training. Simulation provides training in low-frequency, high-risk clinical situations. A longitudinal simulation curriculum could provide consistent training for residents to develop these essential skills.

**Methods:**

We utilized Kern's 6 steps of curriculum development. A needs assessment identified content gaps. We implemented a 3-year, 9-case resuscitation simulation curriculum for pediatric residents targeting shock (septic, hypovolemic, obstructive), arrhythmias (supraventricular tachycardia, ventricular fibrillation, asystole), and respiratory failure (with bradycardia, seizures, hypoventilation). Each year, participants completed 3 simulation cases over 3 hours utilizing high-technology manikins. Patients ranged from 10 days to 10 years. We assessed the curriculum using Kirkpatrick's model, including reaction data assessing for change in self-reported confidence recognizing and managing targeted clinical events from pre/postsurveys, free-text responses noting areas of improved knowledge on postsurveys, and chart review of code events assessing for behavior change if residents led actual codes.

**Results:**

Ninety-two pediatric residents completed the curriculum. Participation was associated with improved confidence recognizing and managing targeted clinical events (pre: median 3-4, interquartile range [IQR] 3-5; post: 4-5, IQR 4-5; *P* < .05). Themes of lessons learned included increased knowledge in clinical management, crisis resource management skills, and local resources. Residents were more likely to assume the code leader role in actual patient codes postcurriculum (10% pre vs 40% post).

**Discussion:**

Participation in the curriculum was associated with increased resident confidence recognizing and managing targeted content, improved knowledge, and greater resident participation as code leaders in actual codes.

## Educational Objectives

By the end of this activity, learners will be able to:

1. Formulate a differential diagnosis for a pediatric patient presenting with shock, arrhythmias, and respiratory failure.
2. Demonstrate the initial management of a pediatric patient presenting with shock, arrhythmias, and respiratory failure.
3. Demonstrate the steps of obtaining vascular access (including intravenous and intraosseous access), airway management (including bag-mask ventilation and intubation), and use of a defibrillator.

## Introduction

The Accreditation Council for Graduate Medical Education requires pediatric residents be able to provide initial management to severely ill patients by the end of training.^1^ However, pediatric cardiorespiratory events are low-frequency, high-risk events with high morbidity and mortality,^2^ and residents may not have reliable training in these events during clinical rotations.^3^ As a result, residents often lack the confidence, knowledge, and skills to manage severely ill patients, including during codes.^4^ Although Pediatric Advanced Life Support (PALS) and Basic Life Support (BLS) training are important components of this training, evidence suggests that these courses alone are insufficient, with knowledge gaps and skill deterioration over time. Supplemental, repetitive resuscitation training is therefore essential^4-6^ to provide residents with predictable opportunities to develop and maintain these skills within pediatric residency.

Simulation-based medical education (SBME) is an effective educational tool to improve learning and patient care.^7-9^ SBME provides training in low-frequency, high-risk situations to supplement clinical training. Kolb's experiential learning theory^10^ provides a framework to support the use of simulation to increase training in pediatric code events. Trainees are immersed in a concrete experience during the simulation that they reflect upon and conceptualize during debriefs and actively apply to future encounters.

While SBME is effective, it can be resource-intensive,^11,12^ and resources vary widely across residencies, potentially limiting feasibility.^13^ Having comprehensive resources for implementing SBME for managing severely ill patients for pediatric residents can help address barriers.^13^ Previous studies have demonstrated the benefits of standardized longitudinal simulation curricula, but materials from many of these curricula are not readily available for other programs to utilize.^14-16^ Many focus on fellows rather than residents^15^ or on select topics such as procedural training.^17,18^ While there are numerous single simulation cases^19-23^ and multicase workshops^24^ addressing pediatric resuscitation available on *MedEdPORTAL*, there is a paucity of comprehensive curricula for programs looking to implement a complete curriculum across a 3-year residency program focused on both clinical and procedural skills. Our goal was to develop a widely accessible 3-year pediatric resuscitation simulation curriculum targeting the care of severely ill patients as a teaching activity and assess its impact on pediatric resident confidence, knowledge, and participation in code events.

## Methods

### Activity Design and Development

We utilized Kern's 6 steps for curriculum development when designing the curriculum. We conducted a needs assessment survey of residents, program leadership, and core faculty and reviewed trends in codes to identify content to address. We identified a need for additional training in and confidence managing common pediatric resuscitation skills with a focus on initial recognition and management and common reasons for code events, including shock, cardiac arrhythmias, and respiratory failure. Based on that, and in alignment with PALS core content, we identified a need to increase residents’ ability to diagnose and manage pediatric code events related to those presentations and perform related procedures.

### Curricular Context and Learner Preparation

We developed a 3-year, 9-case resuscitation simulation curriculum for pediatric residents (Appendix A). Learning objectives focused on 3 core concepts (shock, arrhythmia, and respiratory failure) with an emphasis on differentiating clinical presentations and initial management. Key interventions aligned with PALS algorithms and local content expert guidance, with an emphasis on the first minutes of care. The development team included faculty in pediatric hospital medicine, emergency medicine, and critical care, as well as chief residents. We piloted cases on a subgroup of residents and implemented modifications based on input received.

The cases and learning objectives included:

1. Shock
  • Differentiate between septic, hypovolemic, and obstructive shock
  • Demonstrate the initial management of the patient in septic shock, including providing appropriate fluid management, glucose supplementation, and antibiotics
  • Demonstrate the initial management of the patient in hypovolemic shock, including providing appropriate fluids and managing hypoglycemia
  • Demonstrate initial management of obstructive shock from coarctation of the aorta, including intravenous/intraosseous access, judicious fluid management, prostaglandin E1, and inotropic support
  • Demonstrate steps of obtaining vascular access, including intravenous and intraosseous
2. Arrythmia
  • Correctly identify arrhythmias, including differentiating supraventricular tachycardia, ventricular fibrillation, and asystole
  • Identify differential diagnosis for cause of cardiac arrest (H's & T's)
  • Demonstrate the stepwise algorithm for managing supraventricular tachycardia, ventricular fibrillation, and asystole
  • Demonstrate appropriate use of the defibrillator to perform synchronized cardioversion
3. Respiratory failure:
  • Verbalize the differential for respiratory failure, including respiratory depression from medications and lower respiratory infections
  • Demonstrate initial management of a patient with hypoxia and symptomatic bradycardia, including initiation of cardiopulmonary resuscitation and use of epinephrine
  • Demonstrate initial management of respiratory failure, including effective bag-mask ventilation and reversal agents when appropriate

The curriculum used a spiral design, with each year covering 3 core concepts (shock, arrhythmia, and respiratory failure) to reinforce key learning objectives, while varying etiologies each year to broaden diagnostic exposure. We covered the same procedures annually (vascular access, including intravascular and intraosseous access with shock, defibrillator use with arrhythmias, and airway management, including bag-mask ventilation and intubation for respiratory failure) for repeat practice.

We used a single annual session to allow sufficient time to cover 3 cases, reinforce connections between cases, and promote deeper discussion during repeat debriefs. This was also more feasible to schedule rather than pulling residents from multiple rotations. The annual structure supported reinforcement of key concepts to promote longer-term retention.

All residents were PALS and BLS certified prior to participating.

### Facilitator Training

At baseline prior to curriculum implementation, facilitators participated in a peer-review process, including an asynchronous course on best practices in simulation facilitation. Members of our simulation leadership team reviewed facilitators’ debriefs every 2 years using Debriefing Assessment for Simulation in Healthcare^25^ as part of the routine reassessment of facilitators to ensure high-quality, standardized simulation facilitation across the curriculum. We also provided a guide outlining key points to highlight in alignment with objectives (Appendix B).

The Medical College of Wisconsin Institutional Review Board determined that this project met the criteria for exemption on February 27, 2017 (PRO00027514).

### Equipment/Environment

#### Simulation cases

We conducted cases in our simulation center rooms designed to align with the clinical setting. We utilized Laerdal SimJunior and SimBaby manikins placed on hospital beds. Each room included the required equipment (Appendix C) next to the bed. A computer above the bed displayed vitals and cardiac rhythms. A separate screen displayed the requested laboratory tests/imaging via PowerPoint (Appendix D).

#### Procedural training

Each case had an associated procedural station that occurred in a conference room adjacent to the simulation center. Procedure equipment is outlined in Appendix C.

#### Personnel

Two facilitators participated with each group of 3 to 4 residents. One facilitator controlled the manikin from behind a 1-way mirror. The other facilitator assumed the role of a nurse embedded participant in the room, assisted with tasks outside the resident's scope of practice, and provided historical information. Both facilitators participated in debriefs and procedure stations.

#### Implementation

The sessions were held monthly for 3 hours. Each resident in our program participated in 1 session per year and completed the 9 cases over their 3-year training. Residents attended sessions based on rotations most amenable to being absent. Residents self-selected roles during cases, and facilitators encouraged them to rotate roles for each case.

#### Prebrief^26^

One facilitator led a prebrief prior to the cases (Appendix E), which included an overview of the session objectives, an orientation to the equipment, and a fiction contract.

#### Cases

Residents were divided into 2 groups intended to have a mix of resident years in each group. The groups rotated through 3 cycles of a 10-minute case, a 20-minute debrief, and a 15-minute procedure.

Facilitators followed a guide outlining key steps for the case (Appendix B). They informed participants of the patient's setting, chief complaint, and background. We provided the requested laboratory tests on PowerPoint slides (Appendix D). A facilitator behind a 1-way mirror adjusted vitals and manikin settings as outlined in Appendix B. Vitals and rhythms were projected onto a screen above the bed. Each case progressed to the point of handoff to the accepting team. The case did not include any documentation or order entry.

A debrief occurred after each case in an adjacent conference room. Facilitators used the PEARLS framework^27^ to support learner reflection and engagement. Facilitators used materials in Appendix B to facilitate discussions, standardize content, and align with learning objectives. Facilitators received a tool to assist in prioritizing discussion points during debriefs (Appendix G). The facilitators introduced and demonstrated the targeted procedures (Appendix B). The participants practiced each skill using supplies provided (Appendix C). The cycle of case, debrief, and procedure skill was repeated 3 times to complete 3 cases and 5 procedures.

#### Assessment

Residents completed pre- and postassessments immediately before/after the sessions (Appendix F) via a QR code to an electronic survey. The study team developed the assessments, with the procedure-based questions adapted from Accreditation Council for Graduate Medical Education survey items. The assessments were piloted and refined based on pilot feedback.

We assessed resident self-reported confidence recognizing and managing pediatric shock, arrhythmias, and respiratory failure and performing targeted procedures prior/post the session (5-point Likert scale assessing agreement with the statement that they feel confident recognizing and managing targeted content and performing targeted procedures, 1 = strongly disagree, 5 = strongly agree). Residents assessed if sessions were a valuable learning experience and if they received feedback that helped them learn (1 = strongly disagree, 5 = strongly agree). Wilcoxon signed-rank test assessed for changes in confidence. Descriptive statistics analyzed learners’ perceptions of the educational experience.

#### Knowledge

The survey included a free-text response asking participants to indicate an element of knowledge gained during sessions. We conducted a qualitative thematic analysis to identify themes in subjective lessons learned. Two investigators independently reviewed responses and developed initial codes. Investigators compared codes, discussed discrepancies, and reconciled differences through consensus. Themes and subthemes were identified. Representative comments were selected to illustrate key themes. Data analysis continued until thematic sufficiency was reached.

#### Behavior

Senior residents at our institution could serve as code leaders on evenly numbered days. Critical care fellows led on odd days. At baseline, residents rarely assumed this role, citing a lack of confidence and prior exposure as primary limitations. We assessed the percentage of resident-led codes on evenly numbered days before and after the implementation of the curriculum to assess if the curriculum was associated with increased resident participation in code events using chi-square testing. All codes included documentation of who led them. We assessed resident participation as code leader by reviewing charts of codes. Baseline data included the time after the role began but before curriculum implementation. We collected postimplementation data after 3 years of curriculum implementation, so seniors had completed the full 3-year cycle.

## Results

We completed 3 full cycles of the 3-year curriculum with 92 pediatric residents participating in all 9 cases. Thirty-five faculty members used the curriculum. We administered the surveys before and after the sessions as a required activity with a 100% response rate.

### Reaction

Participation in the curriculum was associated with improved confidence managing targeted clinical events and procedural skills (Table 1).

**Table 1.**
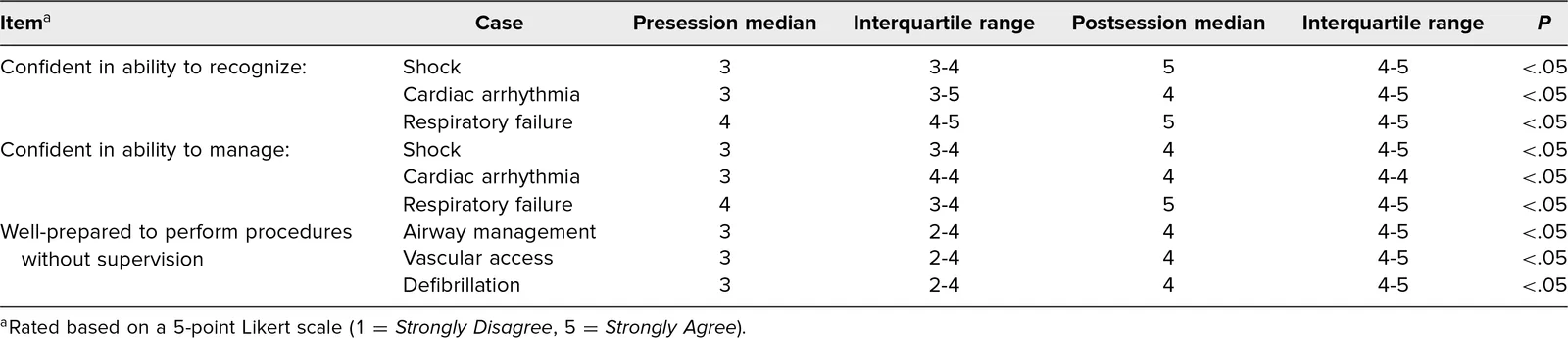
Pediatric Resident Self-Reported Confidence Pre- and Post 3-Year Resuscitation Simulation Curriculum Assessed Via Pre- and Postsurveys (*N* = 92)

Confidence recognizing shock increased from a preintervention median of 3 (interquartile range [IQR], 3-4) to 5 (IQR, 4-5), cardiac arrhythmia from 3 (IQR, 3-5) to 4 (IQR, 4-5), and respiratory failure from 4 (IQR, 4-5) to 5 (IQR, 4-5) (all *P* < .05). Confidence managing shock increased from 3 (IQR, 3-4) to 4 (IQR, 4-5), cardiac arrhythmia from 3 (IQR, 4-4) to 4 (IQR, 4-4), and respiratory failure from 4 (IQR, 3-4) to 5 (IQR, 4-5) (all *P* < .05). Learners reported greater preparedness to perform procedures without supervision, with confidence in airway management increasing from 3 (IQR, 2-4) to 4 (IQR, 4-5), vascular access from 3 (IQR, 2-4) to 4 (IQR, 4-5), and defibrillation from 3 (IQR, 2-4) to 4 (IQR, 4-5) (all *P* < .05).

All residents reported that it was a valuable learning experience and that feedback was valuable to their learning (4-5 on a 5-point Likert scale).

### Subjective Knowledge Gained

Themes of lessons learned included increased knowledge about clinical management, crisis resource management skills, and local resources (Table 2).

**Table 2.**
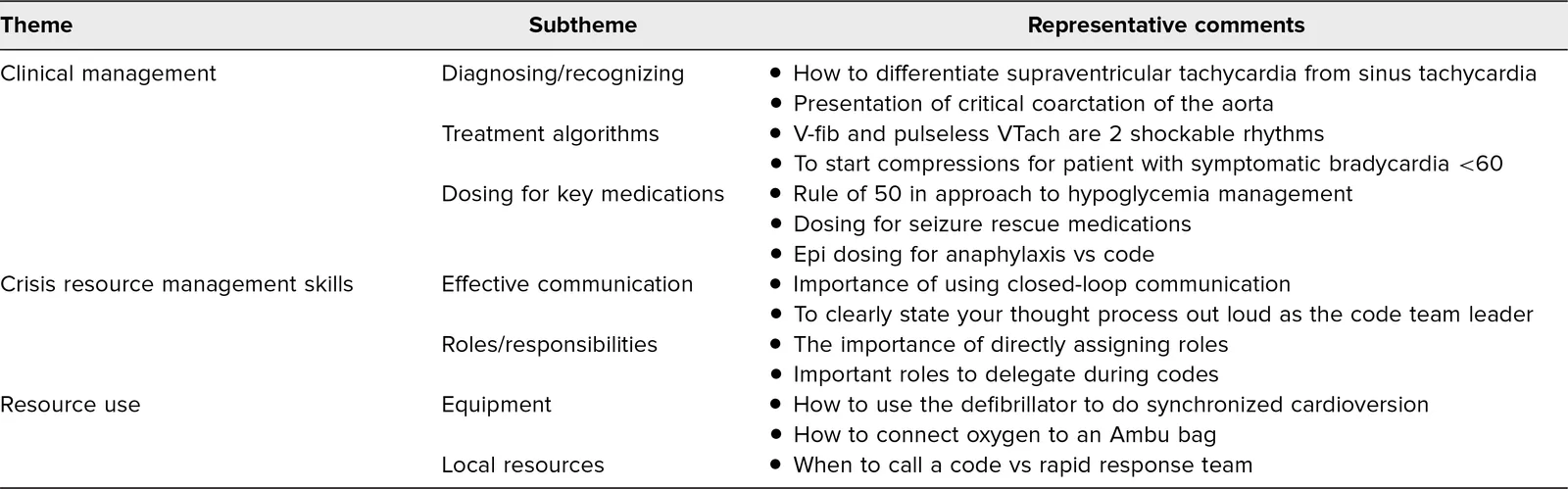
Themes of Lessons Learned From Participating in 3-Year Resuscitation Simulation Curriculum Assessed via Postsession Surveys (*N* = 92)

### Behavior Change

We reviewed 60 even-day codes, 30 precurriculum and 30 postcurriculum implementation. Residents were more likely to lead codes following curriculum implementation (10% [*n* = 3] precurriculum implementation vs 40% [*n* = 12] postcurriculum implementation, *P* < .05).

## Discussion

We successfully implemented a 3-year comprehensive pediatric resuscitation simulation curriculum targeting the care of severely ill patients. Participation in the curriculum was associated with improved resident confidence in recognizing and managing targeted content; self-reported knowledge gained related to clinical management steps, crisis resource management skills, and local resources; and increased resident participation in patient code events.

While we recognize that numerous individual pediatric simulation cases are available, we aimed to share a comprehensive curriculum that could be implemented over a 3-year residency and incorporate both clinical and procedural skills. The spiral design allowed for increased training in different scenarios while also having consistent annual training in key concepts and targeted procedures. Cases could also be used by programs individually to supplement current simulation curricula.

We learned various lessons. We noted a range of baseline performance and found it beneficial to include opportunities to extend cases when participants progressed rapidly and cues to interject when groups were off track (Appendix B). This provided ways to meet individual needs while ensuring we met the intended learning objectives. For example, in a case addressing hypoglycemia, the embedded participant had scripting if a glucose had not been obtained to note how sleepy the patient appeared and request if any additional testing was needed. This allowed learners to reliably identify and manage hypoglycemia. If prompting was required, it was addressed in the debrief regarding indications for obtaining a point-of-care glucose test.

We aimed to ensure residents assumed their actual clinical role. It was beneficial to have an embedded participant facilitator to take on tasks outside the residents’ scope, thereby maintaining their resident role. Residents self-selected roles during cases, such as leader or airway management. Although some groups had 4 residents, so not all residents participated in the code leader role annually, most groups had only 3 residents, so everyone served as code leader throughout their 3 years. We also consistently discussed the role during debriefs to ensure key elements were addressed.

During initial implementation, some participants felt the content was not applicable if they were not pursuing a career in critical care or emergency medicine. We therefore added prebrief content to discuss the application to a broader range of careers, including primary care. Addressing this proactively helped gain buy-in, regardless of future careers.

The initial pilot debriefs did not always focus on intended learning objectives. We therefore expanded the facilitator guide (Appendix B) to provide key content to standardize discussions while allowing flexibility for adaptation based on group performance. We also provided facilitators with a document to take notes on observations to organize the discussion (Appendix F).

We intentionally varied the case location to include both common and atypical locations. This allowed discussion on different approaches based on location (eg, calling a code in the emergency department vs cafeteria).

Although we used high-technology manikins, the curriculum could be adapted to lower-technology resources based on local availability. Displaying vital signs was particularly important to the learning experience to identify changes in patient status. At times, learners required verbal prompting to correctly interpret examinations even on high-technology manikins. The curriculum could therefore likely be conducted using lower-technology manikins, with facilitators verbalizing key examination findings.

There are several limitations to this study. Knowledge data come from open-ended subjective questions that may not reflect sustained improvements in knowledge and reflect what the participants subjectively shared they learned, rather than responding to specific knowledge questions. Based on the comments shared, we felt that the identified themes aligned with the session's objectives. For example, many participants did not know cardiopulmonary resuscitation should be initiated for heart rates less than 60 with poor perfusion despite ventilation, which was a clinically significant fact in alignment with the session's objectives.

Our resident code leader role may not be available at other institutions, which could impact generalizability. Increased participation in leading codes may reflect comfort participating in urgent events in general, which could still be of benefit to institutions without that specific structure.

This study took place at a single institution. Some content addressed logistics specific to our institution, such as when to call a code vs a rapid response. The cases could be easily modified to incorporate local practices at other institutions. We included guidance on where those modifications could be considered.

Next steps include assessing for improvements in resident performance during codes following participation in the curriculum and tracking the time to completion of critical action steps during cases.

This 3-year pediatric resident resuscitation simulation curriculum is an effective way to provide reliable training in high-yield, low-frequency, high-risk pediatric events in the care of a critically ill child. It is a valuable addition to residency training, meeting the requirement to prepare residents to recognize and provide initial management for severely ill patients by the end of training.

## Appendices


Simulation Scenario.docxDebriefing Guide.docxEquipment.docxSupplementary Labs and Imaging.pptxSimulation Prebrief Sample Template.docxPre- and Postsession Learner Survey.docxFacilitator Observation Form.docx
*All appendices are peer reviewed as integral parts of the Original Publication.*
