# Supplementary material for: Comprehensive 3-Year Pediatric Resident Resuscitation Simulation Curriculum on the Care of the Severely Ill Pediatric Patient
Source: MedEdPORTAL. 2026 Nov 6;22:11650. doi: 10.15766/mep_2374-8265.11650 (PMC13741050; doi:10.15766/mep_2374-8265.11650)
Supplement: Supplementary file 1 [file mep_2374-8265.11650-s001.zip › A. Simulation Scenario.docx]

| **Appendix A: *MedEdPORTAL* Simulation Case Template**  **Appendix A: *MedEdPORTAL* Simulation Case**  **SIMULATION CASE TITLE:** Case 1-Shock-Distributive/Septic  **AUTHORS:** Amanda Rogers  **LEARNER AUDIENCE:** Pediatric residents | |
| --- | --- |
| **PATIENT NAME:** Sarah Smith  **PATIENT AGE:** 5-years-old  **CHIEF COMPLAINT:** fever and fussiness  **PHYSICAL SETTING:** Emergency department | |
|  | |
| **Brief Narrative Description of Case** | 5-year-old previously healthy female presented to the Emergency Department with fever and fussiness. The learners will need to recognize and appropriately manage a patient in distributive shock from sepsis and hypoglycemia including fluid resuscitation, glucose supplementation, and empiric antibiotics. |
| **Primary Learning Objectives** | 1. Differentiate the differential diagnosis of shock with emphasis on the categories of shock (hypovolemic, distributive, cardiogenic, and obstructive)  2. Demonstrate the initial management of the patient in septic shock including providing appropriate fluid management, glucose supplementation, and antibiotics  3. Demonstrate steps of obtaining vascular access including intravenous and intraosseous |
| **Critical Actions** | 1. Assess ABCs, place monitors and assign roles for participation 2. Recognize that the child is presenting in shock 3. Attempt to obtain IV access to initiate fluids 4. When unable to obtain IV access, obtain IO access 5. Initiate fluid resuscitation with push pull bolus 6. Recognize hypoglycemia and treat appropriately (IV [5cc/kg of D10 or 10cc/kg of D5] or oral [dextrose gel]) 7. Recognize concern for sepsis as etiology of shock 8. Initiate empiric antibiotic therapy 9. Recognize that the patient is not responsive to fluids and initiate vasoactive agents 10. Determine appropriate disposition and provide handoff to the accepting team |
| **Learner Preparation or Prework** | Prebrief is completed prior to the cases to orient learners to the simulation (appendix F). All participants are Pediatric Advanced Life Support (PALS) and Basic Life Support (BLS) certified prior to participating in the curriculum. PALS algorithm code cards should be available to learners prior to the start of the cases. |

| Initial Presentation | | | |
| --- | --- | --- | --- |
| **Initial Vital Signs** | Temperature: 39.5^o^ Celsius  Respiratory Rate: 25  Oxygen saturation: 95% on room air  Heart rate: 180  Blood pressure: 100/70  Capillary refill time: < 1 second  Pulses: Bounding  Weight 20kg | | |
| **Overall Setting and Appearance** | The case takes place in an Emergency Department exam room. The patient is lying on a hospital bed. The patient is alert and moaning. The nurse is at the bedside. No IV access is in place. There is oxygen with tubing on the wall. Equipment is available on the table next to the bed. (see appendix C) | | |
| **Standardized Participants (and Their Roles in the Room at Case Start)** | The facilitator in the room with the patient is playing the role of the patient’s nurse. They state that they are the nurse caring for the patient and they are worried about the patient’s heart rate. They share that the mom has just stepped out to attend to her other children but that the nurse obtained some additional history from the mom if the residents have any questions. No IV access is in place. | | |
| **HPI** | To be provided to learners at start of case: The patient was recently placed in the ED room and has not been examined by a provider yet. The nurse is concerned about the patient’s condition upon initial assessment and calls the residents to assess. The ED was slow that night and so all the residents went in the room together to assess. The learners have just arrived to the room.  HPI: Sarah is a 5-year-old previously healthy female presenting to the emergency department with fever and fussiness. She was well at baseline until yesterday when she was less playful than normal. This morning when she woke up she was fussy and felt warm. She is not interested in eating or drinking today.  To be provided if asked: She had a temperature of 103 this morning. She has not urinated since last night. She has not been interested in eating or drinking today. She is more sleepy than normal. | | |
| **Past Medical/Surgical History** | **Medications** | **Allergies** | **Family History** |
| Full term. Recurrent ear infections. Otherwise healthy | None | None | Lives with mother and father. No reported ill children at home. |
| **Physical Examination** | | | |
| **General** | Alert, moaning, moving all extremities | | |
| **HEENT** | Sunken eyes, no tears, dry mucous membranes, nose and neck normal | | |
| **Neck** | Supple | | |
| **Lungs** | Clear to auscultation, good air movement | | |
| **Cardiovascular** | Tachycardic, normal rhythm, no murmurs, bounding pulses, cap refill <1s, warm extremities | | |
| **Abdomen** | Soft, nontender, non-distended, no hepatosplenomegaly | | |
| **Neurological** | Alert, cries with exam, moving all extremities | | |
| **Skin** | No rashes | | |
| **GU** | Normal | | |
| **Psychiatric** | Normal | | |

| Instructor Notes - Changes and CASE Branch Points | | |
| --- | --- | --- |
| **Intervention / Time Point** | **Change in Case** | **Additional Information** |
| Learners enter room, assess ABCs, place monitors and assign roles for participation | Recognize patient in shock |  |
| Administer supplemental oxygen | Oxygen saturation increases to 98% |  |
| Ask nurse to obtain peripheral IV access | Nurse reports unable to obtain |  |
| Obtain IO access | IO access successfully obtained | If IO not placed and/or fluids not initiated, heart rate should continue to increase and blood pressure should decrease to 70/30 |
| 1^st^ 20cc/kg push pull bolus of normal saline or lactate ringers | Heart rate improves to 170 | Nurse should ask how bolus should be administered to prompt discussion of bolus rates and consideration of push-pull method |
| Recognize concern for hypoglycemia and request glucose check | Nurse reports glucose is 40 | If learners do not identify hypoglycemia, nurse can prompt by noting how sleepy child is and asking if anything should be done to address this |
| Administer glucose (IV [5cc/kg of D10 or 10cc/kg of D5] or oral [dextrose gel] if IV access not available at the time) | Patient becomes more alert | If repeat requested, glucose is now 100 |
| Obtain cultures and initiate empiric antibiotics | No change in status |  |
| 2^nd^ 20cc/kg push pull bolus of normal saline or lactate ringers | No change in status |  |
| 3^rd^ 20cc/kg push pull bolus of normal saline or lactate ringers | No change in status |  |
| Initiation of vasoactive medication | Heart rate improves to 120  Capillary refill 2 seconds  Pulses 2+ |  |
| If labs/imaging requested at any point in case | Provide labs/imaging (appendix D) |  |
| Determine disposition plan and provide handoff to accepting team |  |  |

**Ideal Scenario Flow**

*The learners enter the room to find a patient in distributive shock from sepsis. They assess airway, breathing, and circulation, apply monitors, and assign roles for participants. They initiate supplemental oxygen and request IV access for fluid resuscitation. When the facilitator assuming the role of the nurse states that they are not able to get IV access after three attempts, the learners obtain intraosseous access which will be successful and give 20ml/kg bolus normal saline or lactated ringer’s via push/pull with mild improvement in heart rate. While bolus is in progress, they obtain a point of care glucose which is 40. They address the hypoglycemia with either IV [5cc/kg of D10 or 10cc/kg of D5] or oral [dextrose gel] depending on if access is available at the time. They recognize improved but persistent tachycardia and provide a 2^nd^ and 3^rd^ bolus. They administer empiric antibiotics for suspected septic shock. The patient will not improve with the additional fluid resuscitation. They will then initiate vasoactive medication with subsequent improvement in heart rate and perfusion. Learners should determine appropriate disposition and provide handoff to the accepting team*

**Anticipated Management Mistakes**

1. *Failure to identify hypoglycemia: Many of the learners did not obtain a point of care glucose early in the case. Some groups identified the hypoglycemia when they obtained a basic metabolic panel later in the case. If groups did not ever request a glucose test, we found it helpful to have the nurse prompt the team by noting the patient’s sleepiness and lack of oral intake and ask if any additional testing should be done. This typically prompted the test to be obtained so the group could still walk through the management of hypoglycemia*
2. *Failure to recognize and sufficiently treat compensated shock: We intentionally have the patient start in compensated shock with a normal blood pressure to highlight the need to recognize and urgently manage patients at that stage of the case. Some groups were inappropriately reassured by the normal blood pressure despite significant tachycardia and signs of warm shock. If needed we would have the case progress to uncompensated shock by having the blood pressure decrease. We would then address this in the debrief of the order in which vital sign changes are seen in pediatric shock and the importance of recognizing and aggressively treating compensated shock before it progresses to uncompensated shock.*
3. *Role delegation: Learners do not typically assign roles for this case due to not aligning with typical code events, which can lead to conflicting communication and missed tasks. We include a discussion of roles and responsibilities in the debrief to assess this element.*

| **Appendix A: *MedEdPORTAL* Simulation Case**  **SIMULATION CASE TITLE:** Case 2-Arrhythmia-Supraventricular tachycardia (SVT)  **AUTHORS:** Amanda Rogers  **LEARNER AUDIENCE:** Pediatric Residents | |
| --- | --- |
| **PATIENT NAME:** Kaylee Cash  **PATIENT AGE:** 8-month-old  **CHIEF COMPLAINT:** Poor feeding and fussiness  **PHYSICAL SETTING:** Acute care hospital room | |
|  | |
| **Brief Narrative Description of Case** | 8-month-old previously healthy female presented as a direct admission to the hospital with fussiness and poor feeding. The learners will need to recognize and appropriately manage a patient with supraventricular tachycardia including vagal maneuvers, adenosine, and synchronized cardioversion. |
| **Primary Learning Objectives** | 1. Correctly identify arrhythmias including differentiating supraventricular tachycardia from sinus tachycardia 2. Demonstrate the stepwise algorithm for managing supraventricular tachycardia 3. Demonstrate appropriate use of the defibrillator to perform synchronized cardioversion |
| **Critical Actions** | 1. Assess ABCs, place monitors and assign roles for participation 2. Recognize concern for supraventricular tachycardia 3. Trial vagal maneuvers including ice the to face 4. Obtain IV access (preferably in right upper extremity) 5. Apply defibrillation pads 6. Trial adenosine (0.1mg/kg rapid bolus, first max dose 6mg) 7. Consider 2^nd^ dose of adenosine (0.2mg/kg rapid bolus, first max dose 12 mg) 8. Recognize development of poor perfusion 9. Consult cardiology and perform synchronized cardioversion (0.5-1 J/kg) 10. Determine appropriate disposition and provide handoff to the accepting team |
| **Learner Preparation or Prework** | Prebrief is completed prior to the cases to orient learners to the simulation (appendix F). All participants are Pediatric Advanced Life Support (PALS) and Basic Life Support (BLS) certified prior to participating in the curriculum. PALS algorithm code cards should be available to learners prior to the start of the cases. |

| Initial Presentation | | | |
| --- | --- | --- | --- |
| **Initial Vital Signs** | Temperature: 37.6^o^ Celsius  Respiratory Rate: 22  Oxygen saturation: 96% on room air  Heart rate: 220  Blood pressure: 110/65  Capillary refill time: 2 seconds  Pulses: 2+  Weight 20kg | | |
| **Overall Setting and Appearance** | The case takes place in an acute care hospital room. There is oxygen with tubing on the wall. The patient is lying on a hospital bed. The patient is alert and crying. No IV access is in place. There is oxygen with tubing on the wall. Equipment is available on the table next to the bed. (see appendix C) | | |
| **Standardized Participants (and Their Roles in the Room at Case Start)** | The facilitator in the room with the patient is playing the role of the patient’s nurse. They state that they are the nurse caring for the patient and they are worried about the patient’s heart rate. They share that the mom has just stepped out to attend to her other children but that the nurse obtained some additional history from the mom if the residents have any questions. No IV access is in place. | | |
| **HPI** | To be provided to learners at start of case:  The patient recently arrived to the hospital as a direct admission from their primary care provider’s office. They have not been examined by a provider yet. The nurse is concerned about the patient’s condition upon initial assessment and calls the residents to assess. The learners have just arrived to the room.  HPI: Kaylee is an 8-month-old previously healthy female presenting to the acute care unit with poor feeding and fussiness. She was well until day of admission when she became fussy and was not feeding well.  To be provided if asked:  She has never had any episodes like this before. She has been otherwise well at baseline. No testing or interventions were done at the primary care provider office prior to arrival | | |
| **Past Medical/Surgical History** | **Medications** | **Allergies** | **Family History** |
| Previously healthy | None | None | Lives with mother, father and 5-year-old brother. No reported ill children at home. |
| **Physical Examination** | | | |
| **General** | Awake, crying | | |
| **HEENT** | Moist mucous membranes | | |
| **Neck** | Supple | | |
| **Lungs** | Clear to auscultation bilaterally. No wheezes or crackles. | | |
| **Cardiovascular** | Tachycardic, normal heart sounds without murmur, rub or gallop. Pulses 2+, cap refill 2 seconds | | |
| **Abdomen** | Soft, non-tender, non-distended. Normal BS. No HSM. | | |
| **Neurological** | Fussy | | |
| **Skin** | No rashes, no mottling | | |
| **GU** | Normal | | |
| **Psychiatric** | Normal | | |

| Instructor Notes - Changes and CASE Branch Points | | |
| --- | --- | --- |
| **Intervention / Time Point** | **Change in Case** | **Additional Information** |
| Learners enter room, assess ABCs, place monitors and assign roles for participation | Recognize supraventricular tachycardia |  |
| Vagal maneuvers (eg ice to face) | No change in vitals or exam |  |
| Request IV access | Nurse is able to obtain IV access | Nurse should ask if preference on where IV placed to prompt future discussion in debrief on preferred location of IV for adenosine |
| Apply defibrillation pads |  |  |
| 1^st^ dose of adenosine (0.1mg/kg rapid bolus, max first dose of 6mg) | Rhythm briefly changes to asystole in response to adenosine, then returns to SVT |  |
| 2^nd^ dose of adenosine (0.2mg/kg rapid bolus, max first dose of 12mg) | Rhythm briefly changes to asystole in response to adenosine, then returns to SVT |  |
| Patient subsequently becomes less responsive and decompensates with poor perfusion | No longer crying, less interactive  New vitals:  Temperature: 37.6^o^ Celsius  Respiratory Rate: 22  Oxygen saturation: 96% on room air  Heart rate: 220  Blood pressure: 70/30  Capillary refill time: 4 seconds  Pulses: 1+ | If learners are uncertain of subsequent steps, can have nurse prompt them by asking if there is anyone they should call and can provide recommendation from cardiology to consider synchronized cardioversion |
| Consult cardiology and consider synchronized cardioversion (0.5 - 1 J/kg) | Cardiac rhythm changes to normal sinus rhythm. Patient is more alert and calm  New vitals:  Heart rate: 100  Blood pressure: 110/65  Capillary refill time: 3 seconds  Pulses: 2+ |  |
| If labs/imaging requested at any point in case | Provide labs/imaging (appendix D) |  |
| Determine disposition plan and provide handoff to accepting team |  |  |

**Ideal Scenario Flow**

*The learners enter the room to find a patient in supraventricular tachycardia. They assess airway, breathing, and circulation, apply monitors, and assign roles for participants. They should initiate vagal maneuvers. After seeing no changes in vitals, the learner should place an IV, (preferably in the upper extremity), apply pads, and administer adenosine (0.1mg/kg rapid bolus, max first dose of 6mg). No change is seen with the adenosine. Learners can administer a 2^nd^ dose of adenosine (0.2mg/kg rapid bolus, max first dose of 12mg). No change is seen with adenosine. The patient then has subsequent decompensation with decreased level of alertness and hypotension. The learners recognize decompensation and perform synchronized cardioversion (0.5 - 1 J/kg) with subsequent change to sinus rhythm. The learners will contact cardiology, determine disposition plan (acute care vs PICU based on institutional standard) and provide handoff to accepting provider.*

**Anticipated Management Mistakes**

*1. Lack of familiarity on how to perform vagal maneuvers including ice to the face. Many residents are aware of need to perform vagal maneuvers but are unaware of how to specifically do this task. We found it helpful to have ice and a bag available to allow them to demonstrate specific skill including placing ice water in a bag.*

*2. Lack of familiarity with how to administer adenosine: Many residents were aware there were unique aspects of how to give adenosine but were not familiar with how to administer it. We included equipment to demonstrate the process of using a three way stop cock to provide adenosine using the rapid bolus technique in the debrief when needed.*

*3. Lack of familiarity with the use of synchronized cardioversion on defibrillator: Participants typically know when to use synchronized cardioversion but now how to do so on our institution’s device. We have therefore included that as an element of the procedural debrief for this case to focus on the use of that equipment.*

| **Appendix A: *MedEdPORTAL* Simulation Case**  **SIMULATION CASE TITLE:** Case 3-Respiratory failure/bradycardia  **AUTHORS**: Amanda Rogers  **LEARNER AUDIENCE:** Pediatric Residents | |
| --- | --- |
| **PATIENT NAME:** Ben Brown  **PATIENT AGE:** 5-year-old  **CHIEF COMPLAINT:** Respiratory distress  **PHYSICAL SETTING:** Acute care hospital room | |
|  | |
| **Brief Narrative Description of Case** | 5-year-old previously healthy male admitted for respiratory distress develops bradycardia and slow breathing. The learners will need to recognize and appropriately manage a patient with symptomatic bradycardia and respiratory failure including performing CPR and administering epinephrine. |
| **Primary Learning Objectives** | 1. Verbalize the differential for respiratory failure including lower respiratory infection 2. Demonstrate initial management of respiratory failure including effective bag mask ventilation 3. Demonstrate initial management of a patient with hypoxia and symptomatic bradycardia including initiation of CPR and use of epinephrine |
| **Critical Actions** | 1. Assess ABCs, place monitors, and assign roles for participation 2. Recognize bradycardia with a pulse and cardiorespiratory compromise 3. Initiate bag mask ventilation 4. Initiate CPR 5. Give epinephrine (0.01mg/kg 0.1mL/kg 1:10,000) 6. Determine appropriate disposition and provide handoff to the accepting team |
| **Learner Preparation or Prework** | Prebrief is completed prior to the cases to orient learners to the simulation (appendix F). All participants are Pediatric Advanced Life Support (PALS) and Basic Life Support (BLS) certified prior to participating in the curriculum. PALS algorithm code cards should be available to learners prior to the start of the cases. |

| Initial Presentation | | | |
| --- | --- | --- | --- |
| **Initial Vital Signs** | Temperature: 37.6^o^ Celsius  Respiratory Rate: 10  Oxygen saturation: 70% on room air  Heart rate: 50  Blood pressure: 110/70  Capillary refill time: 4 seconds  Pulses: 1+  Weight: 20kg | | |
| **Overall Setting and Appearance** | The case takes place in an acute care hospital room. The patient is lying on a hospital bed. The patient is non-responsive and is taking slow, shallow breaths. The nurse is at the bedside. There is oxygen with tubing on the wall. IV access in in place. Equipment is available on the table next to the bed. (see appendix C) | | |
| **Standardized Participants (and Their Roles in the Room at Case Start)** | The facilitator in the room with the patient is playing the role of the patient’s nurse. They state that they are the nurse caring for the patient and they are worried about the patient’s breathing and heart rate. They share that the mom has just stepped out to attend to her other children but that the nurse obtained some additional history from the mom if the residents have any questions. IV access is in place in the right upper extremity. | | |
| **HPI** | To be provided to learners at start of case:  The learners are part of a resident team starting their day who have not yet examined this patient. The patient was admitted by the night team last night. The team is called by the nurse to come assess due to a change in their clinical status. The learners have just arrived to the room.  HPI: Ben is a previously healthy 5-year-old male who initially presented to the acute care unit in respiratory distress. He was admitted overnight after presenting with three days of increased work of breathing, cough, retractions, and nasal flaring. He was initially doing well on 2L nasal cannula but has had increasing oxygen requirements over the last hour. He has become progressively more pale and lethargic over the last 15 minutes. He also had weak pulses and agonal respirations.  To be provided if asked:  He has not had any recent respiratory treatments. | | |
| **Past Medical/Surgical History** | **Medications** | **Allergies** | **Family History** |
| Full term, previously healthy. No surgical history | Multivitamin | None | Dad has asthma. Grandma with hypertension. Lives with mother and father and 5 siblings. No daycare. |
| **Physical Examination** | | | |
| **General** | Limp, pale, nonresponsive | | |
| **HEENT** | Normocephalic/atraumatic, moist mucous membranes | | |
| **Neck** | Supple | | |
| **Lungs** | Decreased respiratory effort, coarse breath sounds throughout | | |
| **Cardiovascular** | Bradycardic, cap refill 4 seconds, 1+ peripheral pulses | | |
| **Abdomen** | Soft, non-distended, non-tender | | |
| **Neurological** | Not responsive, no spontaneous movements, 2+ reflexes | | |
| **Skin** | Normal | | |
| **GU** | Normal | | |
| **Psychiatric** | Non-responsive | | |

| Instructor Notes - Changes and CASE Branch Points | | |
| --- | --- | --- |
| **Intervention / Time Point** | **Change in Case** | **Additional Information** |
| Learners enter room, assess ABCs, place monitors and assign roles for participation | Recognize hypoxia, bradycardia, and poor perfusion |  |
| Bag mask ventilation | Heart rate initially improves to 70 and saturations improve to 100% |  |
| Continued bag mask ventilation | Heart rate becomes <50 |  |
| Begin CPR |  | If learners do not initiative CPR despite HR <60, can continue to have HR decrease until it is zero |
| Give epinephrine (0.01mg/kg 0.1mL/kg 1:10,000) | Heart rate improves to 110  Continues with no respiratory effort |  |
| Continue bag mask ventilation |  |  |
| If labs/imaging requested at any point in case | Provide labs/imaging (appendix D) |  |
| Determine disposition plan and provide handoff to accepting team |  |  |

**Ideal Scenario Flow**

*The learners enter the room to find a patient in respiratory failure. They assess airway, breathing, and circulation, apply monitors, and assign roles for participants. They should initiate bag mask ventilate and call a code. The learner should recognize the HR is less than 60 with poor perfusion despite adequate ventilation and should then start CPR. The learner should then obtain IV access and administer IV, epinephrine 1:10,000 0.01mg/kg or 0.1 ml/kg with subsequent return to normal sinus rhythm but not normal respiratory effort. The learners should continue bag mask ventilation, consider intubation, and contact the PICU to arrange for transfer.*

**Anticipated Management Mistakes**

1. *Lack of awareness of need to initiate CPR for symptomatic bradycardia: Some learners are not aware of the need to initiate CPR for heart rate <60 with cardiorespiratory compromise. They frequently note the patient has a pulse and therefore CPR is not needed. If CPR is not initiated within a few minutes of the case starting, we typically make the patient’s pulse continue to drop and eventually be pulseless to prompt the learner to initiate CPR. We then discuss in the debrief the management of bradycardia with cardiorespiratory compromise including initiating CPR if the heart rate is <60.*
2. *Use of relevant tools: The learners do not consistently recognize that this case aligns with a PALS algorithm and do not consistently use that tool to aide in the case. If the learners are not using the tool and are not aware of the correct management steps, we have the facilitator provide a PALS card to the team after a few minutes. This also prompts discussion during the debrief of value of using resources such as PALS cards for less common clinical scenarios.*
3. *Focusing on acute change in status and stabilization: Some learners struggle with initiating management of the patient without first fully understanding their clinical diagnoses. This has provided a good opportunity in the debrief to discuss the balance of assessing ABCs and initiating initial steps of stabilization regardless of the underlying issue as well as when to go back and obtain additional history and evaluation.*

| **Appendix A: *MedEdPORTAL* Simulation Case**  **SIMULATION CASE TITLE:** Case 4-Shock: Hypovolemic  **AUTHORS:** Amanda Rogers  **LEARNER AUDIENCE:** Pediatric Residents | |
| --- | --- |
| **PATIENT NAME:** Lucy Luke  **PATIENT AGE:** 8-month-old  **CHIEF COMPLAINT:** Vomiting and diarrhea for 2 days and increased sleepiness for 1 day  **PHYSICAL SETTING:** Emergency department exam room | |
|  | |
| **Brief Narrative Description of Case** | 8-month-old previously healthy female presented to the Emergency Department with vomiting, diarrhea and sleepiness. The learners will need to recognize and appropriately manage a patient in hypovolemic shock with hypoglycemia including fluid resuscitation and glucose supplementation. |
| **Primary Learning Objectives** | 1. Differentiate the differential diagnosis of shock with emphasis on the categories of shock (hypovolemic, distributive, cardiogenic, and obstructive)  3. Demonstrate the initial management of the patient in hypovolemic shock, including providing appropriate fluids and managing hypoglycemia  3. Demonstrate steps of obtaining vascular access including intravenous and intraosseous |
| **Critical Actions** | 1. Assess ABCs, place monitors and assign roles for participation 2. Recognize that the child is presenting in shock 3. Attempt to obtain IV access to initiate fluids 4. When unable to obtain IV access, obtain IO access 5. Initiate fluid resuscitation with push-pull boluses 6. Identify concern for hypovolemia as cause of shock 7. Recognize hypoglycemia and treat appropriately (IV [5cc/kg of D10 or 10cc/kg of D5] or oral [dextrose gel]) 8. Determine appropriate disposition and provide handoff to the accepting tea |
| **Learner Preparation or Prework** | Prebrief is completed prior to the cases to orient learners to the simulation (appendix F). All participants are Pediatric Advanced Life Support (PALS) and Basic Life Support (BLS) certified prior to participating in the curriculum. PALS algorithm code cards should be available to learners prior to the start of the cases. |

| Initial Presentation | | | |
| --- | --- | --- | --- |
| **Initial Vital Signs** | Temperature: 37.8^o^ Celsius  Respiratory Rate: 45  Oxygen saturation: 97% on room air  Heart rate: 180  Blood pressure: 90/60  Capillary refill time: 4 seconds  Pulses: 1+  Weight 10kg | | |
| **Overall Setting and Appearance** | The case takes place in an Emergency Department exam room. The patient is lying on a hospital bed. The patient is alert and moaning. The patient wakes with exam but falls asleep quickly. The nurse is at the bedside. No IV access is in place. There is oxygen with tubing on the wall. Equipment is available on the table next to the bed. (see appendix C) | | |
| **Standardized Participants (and Their Roles in the Room at Case Start)** | The facilitator in the room with the patient is playing the role of the patient’s nurse. They state that they are the nurse caring for the patient and they are worried about the patient’s heart rate and sleepiness. They share that the mom has just stepped out to attend to her other children but that the nurse obtained some additional history from the mom if the residents have any questions. No IV access is in place. | | |
| **HPI** | To be provided to learners at start of case: The patient was recently placed in the ED room and has not been examined by a provider yet. The nurse is concerned about the patient’s condition upon initial assessment and calls the residents to assess. The ED was slow that night and so all the residents went in the room together to assess. The learners have just arrived to the room.  HPI: Lucy is an 8-month-old previously health female who presents to the emergency department with vomiting, diarrhea, and increased sleepiness.  To be provided if asked:  She was well at baseline until 2 days ago when she developed vomiting and diarrhea. She has been afebrile. No blood in stool. She has not been interested in drinking and only took two ounces this morning. She has not had a wet diaper in 8 hours which is unusual for her. She also seems more sleepy today. | | |
| **Past Medical/Surgical History** | **Medications** | **Allergies** | **Family History** |
| Bronchiolitis at age 4 months, admitted for 2 days. No other medical illnesses. No surgeries | Tylenol as needed | None | Lives with mother and father. Attends daycare. No reported ill children at home. |
| **Physical Examination** | | | |
| **General** | Laying on bed, crying/moaning, wakes with exam but falls asleep quickly | | |
| **HEENT** | Sunken eyes, no tears, dry mucous membranes | | |
| **Neck** | Supple | | |
| **Lungs** | Good air movement, clear breath sounds, no wheezes or crackles | | |
| **Cardiovascular** | Tachycardic, normal heart sounds without murmur, rub or gallop. Pulses: 1+, cap refill time 4 seconds | | |
| **Abdomen** | Soft, non-distended, no hepatosplenomegaly | | |
| **Neurological** | Sleepy, wakes with exam but falls back asleep, moves all extremities to pain. | | |
| **Skin** | No rashes | | |
| **GU** | Normal | | |
| **Psychiatric** | Sleepy but arousable | | |

| Instructor Notes - Changes and CASE Branch Points | | |
| --- | --- | --- |
| **Intervention / Time Point** | **Change in Case** | **Additional Information** |
| Learners enter room, assess ABCs, place monitors and assign roles for participation | Recognize patient in shock |  |
| Ask nurse to place peripheral IV | Nurse reports unable to obtain peripheral IV |  |
| Obtain IO access | IO access successfully obtained |  |
| Give 1^st^ 20cc/kg push pull bolus of normal saline or lactate ringers | Heart rate improves to 170 | Nurse should ask how bolus should be administered to prompt discussion of bolus rates and consideration of push pull method |
| Give 2^nd^ 20cc/kg push pull bolus of normal saline or lactate ringers | Heart rate improves to 150 | If learners do not provide additional fluid resuscitation, can have HR increase to 180 and decrease blood pressure to 70/30 |
| Give 3^rd^ 20cc/kg push pull bolus of normal saline or lactate ringers | Heart rate improves to 120  Cap refill <2 seconds  Pulses 2+ | If learners do not provide additional fluid resuscitation, can have HR increase to 180 and decrease blood pressure to 70/30 |
| Recognize concern for hypoglycemia and request glucose check | Nurse reports glucose is 40 | If learners do not identify hypoglycemia, nurse can prompt by noting how sleepy child is and asking if anything should be done to address this |
| Administer glucose (IV [5cc/kg of D10 or 10cc/kg of D5] or oral [dextrose gel] if IV access not available at the time) | Patient becomes more alert and interactive | If repeat requested, glucose is now 100 |
| If labs/imaging requested at any point in case | Provide labs/imaging (appendix D) |  |
| Determine disposition plan and provide handoff to accepting team |  |  |

**Ideal Scenario Flow**

*The learners enter the room to find a patient in hypovolemic shock. They assess airway, breathing, and circulation, apply monitors, and assign roles for participants. They initiate supplemental oxygen and request IV access for fluid resuscitation. When the facilitator assuming the role of the nurse states that they are not able to get IV access after three attempts, the learners obtain intraosseous access which will be successful and give 20ml/kg bolus normal saline or lactated ringer’s via push/pull with mild improvement in heart rate. While bolus is in progress, they obtain a point of care glucose which is 40. They address the hypoglycemia with either IV [5cc/kg of D10 or 10cc/kg of D5] or oral [dextrose gel] depending on if access is available at the time. They recognize improved but persistent tachycardia and provide a 2^nd^ and 3^rd^ bolus with gradual normalization of vitals, perfusion, and mental status. Learners should determine disposition plan (acute care vs PICU based on institutional standard) and provide handoff to accepting provider.*

**Anticipated Management Mistakes**

1. *Failure to identify hypoglycemia: Many of the learners did not obtain a point of care glucose early in the case. Some groups identified the hypoglycemia when they obtained a basic metabolic panel later in the case. If groups did not ever request a glucose test we found it helpful to have the nurse prompt the team by noting the patient’s sleepiness and lack of oral intake and ask if any additional testing should be done. This typically prompted the test to be obtained so the group could still walk through the management of hypoglycemia*
2. *Failure to recognize and sufficiently treat compensated shock: We intentionally have the patient start in compensated shock with a normal blood pressure to highlight the need to recognize and urgently manage patients at that stage of the case. Some groups were inappropriately reassured by the normal blood pressure despite significant tachycardia and signs of warm shock. If needed we would have the case progress to uncompensated shock by having the blood pressure decrease. We would then address this in the debrief of the order in which vital sign changes are seen in pediatric shock and the importance of recognizing and aggressively treating compensated shock before it progresses to uncompensated shock.*
3. *Uncertainly on when and how to administer a push pull bolus: We found learners often knew to administer fluids but were unable to verbalize the rate at which the bolus should be given and earlier in their training did not know how to administer a push pull bolus. We therefore added a prompt for the facilitator representing the nurse to ask the team how they would like the fluid administer and provided the supplies needed to perform a push pull bolus so the team could administer the bolus during the case. If they were uncertain on this skill, we include equipment to demonstrate this in the debrief as needed.*

| **Appendix A: *MedEdPORTAL* Simulation Case**  **SIMULATION CASE TITLE**: Case 5-Arrythmia: Ventricular fibrillation  **AUTHORS:** Amanda Rogers  **LEARNER AUDIENCE:** Pediatric Residents | |
| --- | --- |
| **PATIENT NAME:** John Jones  **PATIENT AGE:** 10-year-old boy  **CHIEF COMPLAINT:** Unresponsive  **PHYSICAL SETTING:** Hospital lobby | |
|  | |
| **Brief Narrative Description of Case** | 10-year-old previously healthy male presented to hospital lobby unresponsive after hit to the chest. The learners will need to recognize and appropriately manage an unresponsive pulseless patient with ventricular fibrillation including CPR and performing defibrillation. |
| **Primary Learning Objectives** | 1. Correctly identify arrhythmias, including ventricular fibrillation 2. Identify differential diagnosis for cause of cardiac arrest (H’s & T’s) 3. Demonstrate the stepwise algorithm for managing ventricular fibrillation 4. Demonstrate appropriate use of the defibrillator to perform defibrillation |
| **Critical Actions** | 1. Assess ABCs, place monitors and assign roles for participation 2. Recognize that the child is presenting with non-responsive cardiac arrest 3. Initiate CPR 4. Apply defibrillation pads 5. Identify rhythm as ventricular fibrillation 6. Perform defibrillation (2J/kg initial dose, 4J/kg repeat) 7. Determine appropriate disposition and provide handoff to the accepting team |
| **Learner Preparation or Prework** | Prebrief is completed prior to the cases to orient learners to the simulation (appendix F). All participants are Pediatric Advanced Life Support (PALS) and Basic Life Support (BLS) certified prior to participating in the curriculum. PALS algorithm code cards should be available to learners prior to the start of the cases. |

| Initial Presentation | | | |
| --- | --- | --- | --- |
| **Initial Vital Signs** | Temperature: 37^o^ Celsius  Respiratory Rate: 0  Oxygen saturation: 50% on room air  Heart rate: 0  Blood pressure: unable to obtain  Capillary refill time: 6 seconds  Pulses: not palpable  Weight: 30 kg | | |
| **Overall Setting and Appearance** | The case takes place in the lobby by the hospital welcome desk. The patient collapsed after being struck in the chest by a baseball at a Little League game across the street from the hospital. Parents brought him immediately to the hospital. The patient is lying on the ground by the welcome desk. The patient is non-responsive, not breathing, and does not have a pulse. A nurse who was nearby is at his side. No IV access is in place. Equipment is available on the table next to the patient. (see appendix C) | | |
| **Standardized Participants (and Their Roles in the Room at Case Start)** | The facilitator in the room with the patient is playing the role of a nurse that was at the scene when the patient arrived. They share that the mom has just stepped out to attend to her other children but that the nurse obtained some additional history from the mom if the residents have any questions. | | |
| **HPI** | To be provided to learners at start of case: The patient just arrived at the hospital welcome desk unresponsive. The learners are residents who were nearby the desk and have just arrived to the area.  HPI: John is a 10-year-old male with a history of asthma who presents to the hospital lobby with unresponsiveness after being hit in the chest with a baseball at a Little League game across the street from the hospital.  To be provided if asked:  He is otherwise healthy with no similar events. Was well at baseline that morning. CPR was initiated at the scene and he was brought immediately to the hospital. Was laid down on the ground near the welcome desk. | | |
| **Past Medical/Surgical History** | **Medications** | **Allergies** | **Family History** |
| Intermittent asthma, has not needed albuterol in weeks. No surgical history. | Albuterol as needed. Immunizations are up to date, none missed. | None | Dad has asthma. Grandma with hypertension. Lives with mother and father and 5 siblings. No daycare. |
| **Physical Examination** | | | |
| **General** | Limp, pale, nonresponsive | | |
| **HEENT** | Normocephalic/atraumatic, moist mucous membranes | | |
| **Neck** | Supple | | |
| **Lungs** | No respiratory effort | | |
| **Cardiovascular** | No palpable pulses or audible heart rate, cool, mottled | | |
| **Abdomen** | soft, non-distended, non-tender | | |
| **Neurological** | Not responsive, no spontaneous movements, 2+ reflexes | | |
| **Skin** | No rashes, mottled | | |
| **GU** | Normal | | |
| **Psychiatric** | Non-responsive | | |

| Instructor Notes - Changes and CASE Branch Points | | |  |
| --- | --- | --- | --- |
| **Intervention / Time Point** | **Change in Case** | **Additional Information** | |
| Learners enter room, assess ABCs, place monitors and assign roles for participation | Recognize patient is pulseless and non-responsive |  | |
| Initiate CPR | Saturations improve to 100% with bagging |  | |
| Request IV access | IV access able to be obtained |  | |
| Apply defibrillator pads/cardiac monitor | Ventricular fibrillation on monitor |  | |
| Recognize ventricular fibrillation |  | If rhythm is not correctly identified, can tell learners that cardiology reviewed the rhythm and is concerned about ventricular fibrillation | |
| Perform defibrillation with 2 J/kg | No change |  | |
| Resume CPR and perform rhythm check at 2 minutes | Ventricular fibrillation on monitor |  | |
| Perform defibrillation 4 J/kg | Rhythm resumes normal sinus  New vitals:  Respiratory Rate: 30  Oxygen saturation: 94% on room air  Heart rate: 120  Blood pressure: 90/60  Capillary refill time: 2 seconds  Pulses: 2+ |  | |
| Resume CPR and perform rhythm check at 2 minutes | Rhythm resumes normal sinus  New vitals:  Respiratory Rate: 30  Oxygen saturation: 94% on room air  Heart rate: 120  Blood pressure: 90/60  Capillary refill time: 2 seconds  Pulses: 2+ |  | |
| Determine disposition plan and provide handoff to accepting team |  |  | |

**Ideal Scenario Flow**

*The learners enter the room to find a patient in ventricular fibrillation. They assess airway, breathing, and circulation, apply monitors, and assign roles for participants. They should initiate CPR and apply defibrillation pads. Recognize v fib on the monitor and defibrillate with 2 J/kg. After the first shock is delivered, there will be no change in vitals from the patient which should prompt the learner to resume CPR and administer a second shock 4 J/kg. If learners quickly reach end of case, can have patient require 2nd shock to convert to normal sinus rhythm. If learners take full time to perform initial shock, can have patient convert to sinus rhythm after initial shock. Learners should determine disposition plan (acute care vs PICU based on institutional standard) and provide handoff to accepting provider.*

**Anticipated Management Mistakes**

1. *Proper CPR techniques: While the learners typically recognize the need to initiate CPR, there is variability in the use of proper technique including correct breaths to compression ratio, sufficient rate/depth of compressions, and use of tools such as stools and backboards. We have found it helpful to take note of these elements to address in the debrief as needed.*
2. *Recognition of v fib: Some learners do not appropriate recognize the rhythm on the monitor. If they verbalize an incorrect rhythm we have found it beneficial to state that cardiology has reviewed the monitor and is concerned about v fib to allow them to work through the intended algorithm. We then address this in the debrief session.*
3. *Use of relevant tools: The learners do not consistently use a PALS card to aide in the case and following the respective algorithm. If the learners are not using the tool, we have the facilitator provide a PALS card to the team after a few minutes. This also prompts discussion during the debrief of value of using resources such as PALS cards for less common clinical scenarios.*

| **Appendix A: *MedEdPORTAL* Simulation Case**  **SIMULATION CASE TITLE:** Case 6-Respiratory failure/Seizure  **AUTHORS:** Amanda Rogers  **LEARNER AUDIENCE:** Pediatric Residents | |
| --- | --- |
| **PATIENT NAME:** Timmy Armstrong  **PATIENT AGE:** 10-month-old  **CHIEF COMPLAINT:** abnormal movements  **PHYSICAL SETTING:** Emergency department | |
|  | |
| **Brief Narrative Description of Case** | 10-month-old previously healthy male presented to the Emergency Department with abnormal movements. The learners will need to recognize and appropriately manage a patient with a seizure and subsequent respiratory failure due to anti-seizure medication. |
| **Primary Learning Objectives** | 1. Demonstrate initial management of patient with active seizure, including airway management and treatment with benzodiazepine 2. Verbalize the differential for respiratory failure including respiratory depression from medications 3. Demonstrate initial management of respiratory failure including effective bag mask ventilation |
| **Critical Actions** | 1. Assess ABCs, place monitors and assign roles for participation 2. Recognize that the child is presenting with a generalized seizure 3. Administer anti-seizure medication (IV lorazepam [0.1 mg/kg, max 2 mg] or intranasal midazolam [0.2 mg/kg, max 10 mg]) 4. Recognize respiratory failure as a result of anti-seizure medication 5. Begin bag mask ventilation 6. Determine appropriate disposition and provide handoff to the accepting team |
| **Learner Preparation or Prework** | Prebrief is completed prior to the cases to orient learners to the simulation (appendix F). All participants are Pediatric Advanced Life Support (PALS) and Basic Life Support (BLS) certified prior to participating in the curriculum. PALS algorithm code cards should be available to learners prior to the start of the cases. |

| Initial Presentation | | | |
| --- | --- | --- | --- |
| **Initial Vital Signs** | Temperature: 37^o^ Celsius  Respiratory Rate: 30  Oxygen saturation: 94% on room air  Heart rate: 160  Blood pressure: 90/60  Capillary refill time: 2 seconds  Pulses: 2+  Weight: 10kg | | |
| **Overall Setting and Appearance** | The case takes place in an emergency department room. The patient is actively seizing with upper and lower extremities with rhythmic tonic-clonic movements. The nurse is at the bedside. There is oxygen with tubing on the wall. Equipment is available on the table next to the bed. (see appendix C) | | |
| **Standardized Participants (and Their Roles in the Room at Case Start)** | The facilitator in the room with the patient is playing the role of the patient’s nurse. They state that they are the nurse caring for the patient and they are worried about the patient’s abnormal movements. They share that the mom has just stepped out to attend to her other children but that the nurse obtained some additional history from the mom if the residents have any questions. No IV access is in place. | | |
| **HPI** | To be provided to learners at start of case: The patient was recently placed in the ED room and has not been examined by a provider yet. The nurse is concerned about the patient’s condition upon initial assessment and calls the residents to assess. The ED was slow that night and so all the residents went in the room together to assess. The learners have just arrived to the room.  HPI: Timmy is a 10-month-old previously healthy male presenting to the emergency department with abnormal movements. He was at his grandmother’s house when he developed shaking of his arms and legs. EMS was called and he came to the emergency department.  To be provided if asked:  Timmy was in good health until 20 min ago when he began to have rhythmic shaking of his upper and lower extremities at grandmother’s house. He was playing in grandmother’s bedroom. When he was quiet for several minutes, grandmother went to check on him and found him on the floor, with shaking of arms and legs. He wouldn’t respond. She called 911. In the meantime, seizure stopped after about 5 minutes. When EMS arrived, patient was sleepy, but breathing without problems. Just prior to arrival at ED, he began seizing again. EMS performed no interventions in route. He is now actively seizing on arrival. | | |
| **Past Medical/Surgical History** | **Medications** | **Allergies** | **Family History** |
| Recurrent acute otitis media and had myringotomy tubes placed. No other medical illnesses or other surgeries. | Tylenol as needed. | None | Lives with mother and father. No daycare. Grandma watches during the week. No reported ill children at home |
| **Physical Examination** | | | |
| **General** | Pale and actively seizing; non-responsive. Upper and lower extremities with rhythmic tonic-clonic movements. | | |
| **HEENT** | Normocephalic/atraumatic, moist mucous membranes | | |
| **Neck** | Supple | | |
| **Lungs** | Shallow and stertorous breathing. Good aeration. No wheezes or crackles. | | |
| **Cardiovascular** | Tachycardic, normal heart sounds without murmur, rub or gallop. Pulses 2+, capillary refill 2 seconds | | |
| **Abdomen** | Soft, non-tender, non-distended. Normal bowel sounds. No hepatosplenomegaly | | |
| **Neurological** | Seizing. Does not follow commands or respond appropriately to voice. | | |
| **Skin** | No rashes, no mottling. | | |
| **GU** | Normal | | |
| **Psychiatric** | Nonresponsive | | |

| Instructor Notes - Changes and CASE Branch Points | | |
| --- | --- | --- |
| **Intervention / Time Point** | **Change in Case** | **Additional Information** |
| Learners enter room, assess ABCs, place monitors and assign roles for participation | Recognize patient with generalized seizure activity | If asked, the nurse can report the shaking has been going on for 5 minutes |
| Administer antiseizure medication  (IV lorazepam (0.1 mg/kg, max 2 mg) or intranasal midazolam (0.2 mg/kg, max 10 mg) | No change in vitals or shaking | No IV access is present at arrival so if IV medication is requested, nurse can inquire how to administer |
| Administer 2^nd^ dose of antiseizure medication  (IV lorazepam [0.1 mg/kg, max 2 mg] or intranasal midazolam [0.2 mg/kg, max 10 mg]) | Seizure activity stops. Patient develops respiratory suppression and hypoxia.  New vitals  Respiratory rate – 0  Oxygen saturation – 70%  HR – 100  BP – 90/60  Cap refill – 2 seconds  Pulses – 2+ |  |
| Initiate bag mask ventilation | Oxygen saturation improves to 100% |  |
| Consider need for intubation, contact neurology |  |  |
| If labs/imaging requested at any point in case | Provide labs/imaging (appendix D) |  |
| Determine disposition plan and provide handoff to accepting team |  |  |

**Ideal Scenario Flow**

*The learners enter the room to find a patient seizing. They assess airway, breathing, and circulation, apply monitors, and assign roles for participants. They should recognize that the patient has been seizing for >5 minutes and give 0.1 mg/kg IV lorazepam or 0.2 mg/kg IN midazolam. After initial anti-seizure medication, the patient continues to seize. The leader should repeat benzodiazepine dosing. After a second dose of benzodiazepine by the learner, the patient will stop seizing and also become apneic with hypoxia. The learner should initiate bag mask ventilation (BMV) which should have increased oxygen saturations. The learner should prepare for intubation if necessary. The learner should consider possible causes for seizure. After 5 minutes of BMV, the learner should recognize that the patient is breathing spontaneously, stop BMV and apply O2 and suctioning. They should page neurology, determine disposition plan (acute care vs PICU based on institutional standard) and provide handoff to accepting provider.*

**Anticipated Management Mistakes**

1. *Familiarity with options for seizure rescue: We identified variable awareness of routes and dosing options for seizure rescue medication. We use this as an opportunity to include in the debrief a review of options in patients with and without IVs and a review of our local institutional guidelines to seizure rescue medications.*
2. *Anticipatory awareness of impact of seizure medications on respiratory status: Some learners were more aware of the possible risk of respiratory depression from seizure medications than others. We used this as an opportunity to discuss this possible impact and the consideration of preemptive planning including awareness of where to find equipment and verbalizing to the full team the possibility of respiratory compromise, especially with repeat dosing.*
3. *Proper technique for bag mask ventilation: We found it valuable to have the same equipment available for performing bag mask ventilation as what is available in our clinical setting and to provide the learners with the opportunity to select the correct mask and attach the oxygen tubing to the bag to practice those skills and identify and gaps to focus on during the debrief.*

| **Appendix A: *MedEdPORTAL* Simulation Case**  **SIMULATION CASE TITLE:** Case 7-Shock: Obstructive from coarctation of the aorta  **AUTHORS:** Amanda Rogers  **LEARNER AUDIENCE:** Pediatric Residents | |
| --- | --- |
| **PATIENT NAME:** Annie Anderson  **PATIENT AGE:** 10-day-old female  **CHIEF COMPLAINT:** fast breathing and poor feeding  **PHYSICAL SETTING:** Emergency Department | |
|  | |
| **Brief Narrative Description of Case** | 10-day-old previously healthy female presented to the Emergency Department with difficulty breathing and poor feeding. The learners will need to recognize and appropriately manage a patient with obstructive shock due to coarctation of the aorta including fluid management, cardiac consultation, and consideration of prostaglandins and inotropic support. They will also need to recognize and manage hypoglycemia. |
| **Primary Learning Objectives** | 1. Differentiate the differential diagnosis of shock with emphasis on the categories of shock (hypovolemic, distributive, cardiogenic, and obstructive)  3. Demonstrate initial management of obstructive shock from coarctation of the aorta including IV/IO access, judicious fluid management, prostaglandin E1, and inotropic support  3. Demonstrate steps of obtaining vascular access including intravenous and intraosseous |
| **Critical Actions** | 1. Assess ABCs, place monitors and assign roles for participation 2. Recognize that the child is presenting in shock 3. Attempt to obtain IV access to initiate fluids 4. When unable to obtain IV access, obtain IO access 5. Initiate fluid resuscitation with push pull boluses 6. Recognize and manage hypoglycemia (IV [5cc/kg of D10 or 10cc/kg of D5] or oral [dextrose gel]) 7. Identify concern for coarctation of the aorta as cause of shock 8. Contact cardiology 9. Consider prostaglandins 10. Consider ionotropic support 11. Determine appropriate disposition and provide handoff to the accepting team |
| **Learner Preparation or Prework** | Prebrief is completed prior to the cases to orient learners to the simulation (appendix F). All participants are Pediatric Advanced Life Support (PALS) and Basic Life Support (BLS) certified prior to participating in the curriculum. PALS algorithm code cards should be available to learners prior to the start of the cases. |

| Initial Presentation | | | |
| --- | --- | --- | --- |
| **Initial Vital Signs** | Temperature: 37^o^ Celsius  Respiratory Rate: 75  Oxygen saturation: 94% right upper extremity, 85% lower extremity on room air  Heart rate: 180  Blood pressure: RA 80/40, LA 80/40, RL 30/10, LL 30/10  Capillary refill time: 3 seconds in RUE, 5 seconds in bilateral LE  Pulses: 1+ brachial, femoral pulses not palpable  Weight 4kg | | |
| **Overall Setting and Appearance** | The case takes place in an Emergency Department exam room. The patient is lying on a hospital bed. The patient is pale and has a weak cry. The nurse is at the bedside. There is oxygen with tubing on the wall. Equipment is available on the table next to the bed. (see appendix C) | | |
| **Standardized Participants (and Their Roles in the Room at Case Start)** | The facilitator in the room with the patient is playing the role of the patient’s nurse. They state that they are the nurse caring for the patient and they are worried about the patient’s heart rate and sleepiness. They share that the mom has just stepped out to attend to her other children but that the nurse obtained some additional history from the mom if the residents have any questions. No IV access is in place. | | |
| **HPI** | To be provided to learners at start of case: The patient was recently placed in the ED room and has not been examined by a provider yet. The nurse is concerned about the patient’s condition upon initial assessment and calls the residents to assess. The ED was slow that night and so all the residents went in the room together to assess. The learners have just arrived to the room.  HPI: Annie is a 10-day-old previously healthy full term female presenting to the emergency department with fast breathing and poor feeding  To be provided if asked:  Was well at home until 2 days prior to presentation when developed increased respiratory rate and poor feeding. Is exclusively breast fed. Seems to get tired when feeding. Today had decreased urine output with no wet diaper for 12 hours which prompted them to come to the ED. Has also been more sleepy. | | |
| **Past Medical/Surgical History** | **Medications** | **Allergies** | **Family History** |
| Full term. Normal spontaneous vaginal delivery. Mom was GBS + and adequately treated. No complications with pregnancy or delivery. Normal prenatal care. Home with mom at 2 days. No other medical issues. | None | None | Lives with mother and father. No reported ill children at home |
| **Physical Examination** | | | |
| **General** | Weak, pale, weak cry with painful stimuli, extremities normal appearance | | |
| **HEENT** | Anterior fontanel flat and sunken, sunken eyes, no tears, dry mucus membranes, nose and neck normal | | |
| **Neck** | Supple | | |
| **Lungs** | Tachypnea, symmetric aeration, good air movement | | |
| **Cardiovascular** | Tachycardic, systolic ejection murmur over left precordium radiating to back, no palpable femoral pulses, 1+ right brachial pulse, cap refill 3 seconds in RUE, 5-6 seconds in bilateral LE | | |
| **Abdomen** | Soft, nontender, non-distended, no hepatosplenomegaly | | |
| **Neurological** | Weak cry with painful stimuli | | |
| **Skin** | No rashes, pale, mottled appearance. | | |
| **GU** | Normal | | |
| **Psychiatric** | Non verbal, weak cry with painful stimuli | | |

| Instructor Notes - Changes and CASE Branch Points | | |
| --- | --- | --- |
| **Intervention / Time Point** | **Change in Case** | **Additional Information** |
| Learners enter room, assess ABCs, place monitors and assign roles for participation | Recognize patient is in shock |  |
| Ask nurse to obtain peripheral IV access | Nurse reports unable to obtain |  |
| Obtain IO access | IO access successfully obtained |  |
| Administer 20cc/kg push pull bolus of normal saline or lactate ringers | HR increase to 190 |  |
| Recognize concern for hypoglycemia and request glucose check | Nurse reports glucose is 40 | If learners do not identify hypoglycemia, nurse can prompt by noting how sleepy child is and asking if anything should be done to address this |
| Administer glucose (IV [5cc/kg of D10 or 10cc/kg of D5] or oral [dextrose gel] if IV access not available at the time) | Patient becomes more alert and interactive | If repeat requested, glucose is now 100 |
| Apply monitors and request blood pressure | Blood pressure:  Right arm 80/40  Left arm 80/40  Right leg 30/10  Left leg 30/10 | If 4 extremity blood pressures are not requested, facilitator representing role of nurse should ask where to obtain blood pressure to prompt identification of differential between upper and lower extremities |
| Consider contacting cardiology and initiation of prostaglandins (0.05-0.1 mcg/kg/min) | New vitals  Respiratory Rate: 35  Heart rate: 140  Blood pressure: 85/50  Capillary refill time: 2 seconds  Pulses: 2+ | If team does not take additional steps of calling cardiology or initiating prostaglandins, nursing can prompt learners by asking if there is anyone they would like to contact. |
| If labs/imaging requested at any point in case | Provide labs/imaging (appendix D) |  |
| Determine disposition plan and provide handoff to accepting team |  |  |

**Ideal Scenario Flow**

*The learners enter the room to find a patient in obstructive shock from coarctation of the aorta. They assess airway, breathing, and circulation, apply monitors, and assign roles for participants. They should request IV access for fluid resuscitation. When the facilitator assuming the role of the nurse states that they are not able to get IV access after three attempts, the learners obtain intraosseous access which will be successful. Learners should give a bolus normal saline or LR and reassess. Dexi is obtained which is 40. The learner should treat hypoglycemia (IV [5cc/kg of D10 or 10cc/kg of D5] or oral [dextrose gel]) and repeat the dexi which is 100. Initial labs should be sent. After the IO is placed and the 1^st^ bolus is given, the learner should recognize that the patient does not improve. They should consider alternative causes including cardiac concerns. They will obtain 4 extremity blood pressures and identify concern for coarctation of the aorta. They should contact cardiology and consider initiation of prostaglandin (0.05-0.1 mcg/kg/min) and consider inotropic support. Learners should determine disposition plan based on institutional standard and provide handoff to accepting provider.*

**Anticipated Management Mistakes**

1. *Recognition of obstructive shock from coarctation: Some learners do not recognize the underlying cardiac etiology of the patient’s shock. If the learners do not recognize this within the first few minutes of the case, we have found it beneficial to have the nurse ask about obtaining a blood pressure and if the team has preference on what extremity to use to prompt the recognition of the pulse differential.*
2. *Failure to identify hypoglycemia: Many of the learners did not obtain a point of care glucose early in the case. Some groups identified the hypoglycemia when they obtained a basic metabolic panel later in the case. If groups did not ever request a glucose test we found it helpful to have the nurse prompt the team by noting the patient’s sleepiness and lack of oral intake and ask if any additional testing should be done. This typically prompted the test to be obtained so the group could still walk through the management of hypoglycemia*
3. *Identifying next step in management after concern for coarctation is identified: Some learners are uncertain of next steps after they recognize the concern for possible coarctation. If needed, we have the nurse prompt the team to ask if they would like to call anyone else for help to encourage contacting cardiology. This is a beneficial element of the debrief to discuss the balance of providing initial stabilization to a critically ill child and also using available resources and consulting teams to assist with ongoing management.*

| **Appendix A: *MedEdPORTAL* Simulation Case**  **SIMULATION CASE TITLE:** Case 8-Arrythmia: Asystole  **AUTHORS:** Amanda Rogers  **LEARNER AUDIENCE:** Pediatric Residents | |
| --- | --- |
| **PATIENT NAME:** John Jones  **PATIENT AGE:** 7-year-old  **CHIEF COMPLAINT:** Unresponsive  **PHYSICAL SETTING:** Hospital cafeteria | |
|  | |
| **Brief Narrative Description of Case** | 7-year-old previously healthy male becomes unresponsive at the hospital cafeteria. The learners will need to recognize and appropriately manage an unresponsive pulseless patient with asystole, including CPR and epinephrine. |
| **Primary Learning Objectives** | 1. Correctly identify arrhythmias, including asystole 2. Identify differential diagnosis for cause of cardiac arrest (H’s & T’s) 3. Demonstrate the stepwise algorithm for managing asystole |
| **Critical Actions** | 1. Assess ABCs, place monitors and assign roles for participation 2. Recognize that the child is presenting with asystole 3. Initiate CPR 4. Apply defibrillation pads 5. Recognize asystole on monitor 6. Administer IV epinephrine (0.01mg/kg, (0.1mL/kg of 1:10,000 concentration) 7. Determine appropriate disposition and provide handoff to the accepting tea |
| **Learner Preparation or Prework** | Prebrief is completed prior to the cases to orient learners to the simulation (appendix F). All participants are Pediatric Advanced Life Support (PALS) and Basic Life Support (BLS) certified prior to participating in the curriculum. PALS algorithm code cards should be available to learners prior to the start of the cases. |

| Initial Presentation | | | |
| --- | --- | --- | --- |
| **Initial Vital Signs** | Temperature: 37.6^o^ Celsius  Respiratory Rate: 0  Oxygen saturation: 50% on room air  Heart rate: 0  Blood pressure: unable to obtain  Capillary refill time: 6 seconds  Pulses: not palpable  Weight 20kg | | |
| **Overall Setting and Appearance** | The case takes place in the hospital cafeteria. The patient collapsed in the hospital cafeteria while visiting a family member. The patient is lying on the ground. The patient is non-responsive, not breathing, and does not have a pulse. A nurse who was nearby is at his side. No IV access is in place. | | |
| **Standardized Participants (and Their Roles in the Room at Case Start)** | The facilitator in the room with the patient is playing the role of a nurse that was at the scene when the patient arrived. They share that the mom has just stepped out to attend to her other children but that the nurse obtained some additional history from the mom if the residents have any questions. No IV access is in place. Equipment is available on the table next to the bed. (see appendix C) | | |
| **HPI** | To be provided to learners at start of case:  The patient became unresponsive in the cafeteria after coming to the hospital to visit a family member. The learners are residents who were nearby the cafeteria when it occurred and have just arrived to the area.  HPI: John is a 7-year-old male with a history of intermittent asthma who presents with unresponsiveness. He collapsed in the cafeteria after coming to the hospital to visit a family member. Was witnessed as suddenly becoming unresponsive and falling to the ground.  To be provided if asked:  No other associated symptoms and has never had anything like this previously. Was previously healthy. Had not eaten anything since arriving to the cafeteria. | | |
| **Past Medical/Surgical History** | **Medications** | **Allergies** | **Family History** |
| Intermittent asthma, has not needed albuterol in weeks. No surgical history. | Albuterol as needed | None | Dad has asthma. Paternal uncle had “heart problems”, died at 25 after sudden collapse while playing |
| **Physical Examination** | | | |
| **General** | Limp, pale, nonresponsive | | |
| **HEENT** | Normocephalic/atraumatic, moist mucous membranes | | |
| **Neck** | Supple | | |
| **Lungs** | No respiratory effort | | |
| **Cardiovascular** | No palpable pulses or audible heart rate, cool, mottled | | |
| **Abdomen** | Soft, non-distended, non-tender | | |
| **Neurological** | Not responsive, no spontaneous movements, 2+ reflexes | | |
| **Skin** | Normal | | |
| **GU** | Normal | | |
| **Psychiatric** | Non-responsive | | |

| Instructor Notes - Changes and CASE Branch Points | | |
| --- | --- | --- |
| **Intervention / Time Point** | **Change in Case** | **Additional Information** |
| Learners enter room, assess ABCs, place monitors and assign roles for participation | Recognize patient is pulseless and non responsive |  |
| Initiate CPR | Saturations improve to 100% with bag mask ventilation |  |
| Apply defibrillation pads | Asystole on monitor |  |
| Recognize asystole |  | If rhythm is not correctly identify, can tell learners that cardiology reviewed the rhythm and is concerned about asystole |
| Epinephrine given (0.01mg/kg, (0.1mL/kg of 1:10,000 concentration) | No change |  |
| CPR resumption and repeat epinephrine after 3-5 minutes. |  |  |
| Repeat dose of epinepherine given (0.01mg/kg, (0.1mL/kg of 1:10,000 concentration) | Sinus rhythm on monitor  New vitals:  Respiratory rate – 20  Oxygen saturations – 98%  Heart Rate – 110  BP – 100/60  CRT – 2 sec  Pulses 2+ | . |
| Determine disposition plan and provide handoff to accepting team |  |  |

**Ideal Scenario Flow**

*Provide a detailed narrative description of the way this case should flow if participants perform in the ideal fashion.*

*The learners enter the room to find a patient in cardiac arrest without pulses. They assess airway, breathing, and circulation, apply monitors, and assign roles for participants. The learner should initiate CPR and place defibrillator pads. They should obtain IV access and administer epinephrine 0.01mg/kg (0.1mL/kg 1:10,000). After the 1st epinephrine, there will be no change in vitals and learners are expected to resume CPR and administer a second dose of epinephrine. Advancement of the airway should be considered. After the second dose of epinephrine there is return of sinus rhythm. The learner is expected to transfer the patient to the emergency department and provide handoff to accepting provider.*

**Anticipated Management Mistakes**

1. *Proper CPR techniques: While the learners typically recognize the need to initiate CPR, there is variability in the use of proper technique including correct breaths to compression ratio, sufficient rate/depth of compressions, and use of tools such as stools and backboards. We have found it helpful to take note of these elements to address in the debrief as needed.*
2. *Use of relevant tools: The learners do not consistently use a PALS card to aide in the case and following the respective algorithm. If the learners are not using the tool, we have the facilitator provide a PALS card to the team after a few minutes. This also prompts discussion during the debrief of value of using resources such as PALS cards for less common clinical scenarios.*
3. *Adapting to different locations: Some learners struggle with adapting to being in a different location than the emergency department or hospital unit. We found it beneficial to share the number of actual patient events that occur in atypical locations, discuss how to adapt depending on locations, and review what resources are available based on varied locations.*

| **Appendix A: *MedEdPORTAL* Simulation Case**  **SIMULATION CASE TITLE:** Case 9-Respiratory failure/Hypoventilation  **AUTHORS:** Amanda Rogers  **LEARNER AUDIENCE:** Pediatric residents | |
| --- | --- |
| **PATIENT NAME:** Jacob Thompson  **PATIENT AGE:** 9-year-old  **CHIEF COMPLAINT:** slow breathing  **PHYSICAL SETTING:** Acute care patient room | |
|  | |
| **Brief Narrative Description of Case** | 9-year-old previously healthy male recently admitted following reduction of a leg fracture from the Emergency Department who developed bradycardia and shallow breathing. The learners will need to recognize and appropriately manage a patient with hypoventilation from medication side effects including bag mask ventilation and use of a reversal agent. |
| **Primary Learning Objectives** | 1. Verbalize the differential for respiratory failure including respiratory depression from medications 2. Demonstrate initial management of a patient with hypoventilation from medication overdose including use of reversal agent when appropriate 3. Demonstrate initial management of respiratory failure including effective bag mask ventilation |
| **Critical Actions** | 1. Assess ABCs, place monitors and assign roles for participation 2. Recognize that the child is presenting with respiratory failure 3. Begin bag mask ventilation 4. Recognize concern for hypoventilation from opioid overdose as cause of respiratory failure 5. Administer reversal agent (naloxone [1-5mcg/kg]) 6. Determine appropriate disposition and provide handoff to the accepting team |
| **Learner Preparation or Prework** | Prebrief is completed prior to the cases to orient learners to the simulation (appendix F). All participants are Pediatric Advanced Life Support (PALS) and Basic Life Support (BLS) certified prior to participating in the curriculum. PALS algorithm code cards should be available to learners prior to the start of the cases. |

| Initial Presentation | | | |
| --- | --- | --- | --- |
| **Initial Vital Signs** | Temperature: 37.2^o^ Celsius  Respiratory Rate: 6  Oxygen saturation: 80% on room air  Heart rate: 68  Blood pressure: 100/60  Capillary refill time: < 2 second  Pulses: 2+  Weight 40kg | | |
| **Overall Setting and Appearance** | The case takes place in an acute care hospital room. The patient is lying on a hospital bed. There is a cast on their leg. They are asleep and taking slow shallow breaths. The nurse is at the bedside. There is oxygen with tubing on the wall Equipment is available on the table next to the bed. (see appendix C) | | |
| **Standardized Participants (and Their Roles in the Room at Case Start)** | The facilitator in the room with the patient is playing the role of the patient’s nurse. They state that they are the nurse caring for the patient and they are worried about the patient’s breathing and heart rate. They share that the mom has just stepped out to attend to her other children but that the nurse obtained some additional history from the mom if the residents have any questions. An IV is in place in the right upper extremity. | | |
| **HPI** | Given at start of scenario:  The patient recently arrived to the acute care room as an admission from the emergency department. They have not been examined by a provider yet. The nurse is concerned about the patient’s condition upon initial assessment and calls the residents to assess. The learners have just arrived to the room.  HPI: Jacob is a 9-year-old male with a recent leg fracture who presents on the acute care unit with slow breathing. Patient just arrived to acute care floor for admission for pain control following reduction of leg fracture in ED. On nursing’s initial assessment his monitor was alarming for hypoxia and she noted he was bradypneic so called for assistance.  To be provided if learners ask for additional information: The patient was playing at the park and fell onto his leg. He fractured his leg but sustained no other injuries and had no loss of consciousness. Patient received 2mcg/kg IV fentanyl en route via EMS, then received 0.1 mg/kg of IV midazolam on arrival to the ED. Because of continued pain he received another 2mcg/kg IV fentanyl prior to the sedation. For sedation he received 2mg IV midazolam. Fracture was reduced in the ED and he was subsequently admitted to the floor. Following arrival to the floor, he received 2mcg/kg IV fentanyl for ongoing pain. | | |
| **Past Medical/Surgical History:** | **Medications** | **Allergies** | **Family History** |
| Previously healthy, no issues apart from leg injury | None | No known drug allergies | Mom has diabetes  Brother has asthma |
| **Physical Examination** | | | |
| **General** | Asleep, shallow slow breathing, leg in a cast | | |
| **HEENT** | Moist mucous membranes, pinpoint pupils | | |
| **Neck** | Supple | | |
| **Lungs** | Shallow slow breathing, clear to auscultation bilaterally. No wheezes or crackles. | | |
| **Cardiovascular** | Bradycardic, normal heart sounds without murmur, rub or gallop. Pulses 2+, cap refill 2 seconds | | |
| **Abdomen** | Soft, non-tender, non-distended. Normal bowel sounds. No hepatosplenomegaly | | |
| **Neurological** | Pinpoint pupils, withdraws minimally to painful stimulation Does not follow commands or respond appropriately to voice. | | |
| **Skin** | No rashes, no mottling | | |
| **GU** | Normal exam | | |
| **Psychiatric** | Sleeping | | |

| Instructor Notes - Changes and CASE Branch Points | | |
| --- | --- | --- |
| **Intervention / Time Point** | **Change in Case** | **Additional Information** |
| Learners enter room, assess ABCs, place monitors and assign roles for participation | Recognize patient with respiratory depression |  |
| Supplemental oxygen | No change in vitals or clinical status |  |
| Initiate bag mask ventilation | Oxygen saturation improves to 100% |  |
| Obtain additional history from nurse regarding recent medication administration as noted in HPI |  |  |
| Give naloxone (1-5mcg/kg) | Patient becomes more awake, interactive, crying in pain  New vitals:  Respiratory rate – 18  Oxygen saturations – 97%  Heart Rate – 120  BP – 100/60  CRT – 2 sec  Pulses 2+ | If learners do not identify medication side effect as etiology of symptoms or obtain history regarding prior medications, nursing can prompt team by verbalizing recent medication history and inquire if that is related to symptoms |
| If learners progress through case in <5 minutes, can state 30 minutes has passed and the patient develops recurrent bradycardia, bradypnea and hypoxia | Patient becomes more sleepy, less interactive  New vitals:  Respiratory rate – 6  Oxygen saturations – 80%  Heart Rate – 68  BP – 100/60  CRT – 2 sec  Pulses 2+ | If learners take >5 minutes to progress through case, do not have recurrent symptoms and need for repeat naloxone. Proceed to arranging to transfer to Pediatric Intensive Care Unit and provide handoff to accepting team after initial dose |
| Give 2^nd^ dose of naloxone (1-5mcg/kg) | Patient becomes more awake, interactive, crying in pain  New vitals:  Respiratory rate – 18  Oxygen saturations – 97%  Heart Rate – 120  BP – 100/60  CRT – 2 sec  Pulses 2+ |  |
| If labs/imaging requested at any point in case | Provide labs/imaging (appendix D) |  |
| Determine disposition plan and provide handoff to accepting team |  |  |

**Ideal Scenario Flow**

*The learners enter the room to find a patient with respiratory failure with hypoventilation from the pain medications. They assess airway, breathing, and circulation, apply monitors, and assign roles for participants. They initiate bag mask with improvement in saturations. They administer naloxone (1-5mcg/kg)* *to reverse the opioid induced hypoventilation with improvement in respiratory status. The patient develops recurrent symptoms after ~30 minutes, requiring a repeat dose of naloxone. Following the second dose, the respiratory status normalizes. Learners should determine disposition plan (acute care vs PICU based on institutional standard) and provide handoff to accepting provider.*

**Anticipated Management Mistakes**

1. *Recognize need for bag mask ventilation: Some learners identified hypoxia but did not identify the bradypnea and need for mechanical ventilation. They will continue to increase liters of oxygen but not initiate bag mask ventilation. If needed, can have respiratory rate continue to decrease and eventually become apneic to prompt initiation of bag mask ventilation.*
2. *Recognize etiology of clinical presentation: Some learners do not identify the medication etiology of symptoms. If prompt is needed, the nurse can tell them the medications that had been given even if not asked and inquire if that may be playing a role in their clinical presentation to allow the case to progress.*
3. *Lack of awareness of short half-life of naloxone: Learners were not always aware of the short half-life of naloxone and need to potentially redose or initiate a naloxone drip. If the case was progressing quickly, we built in the patient have recurrent symptoms requiring an additional dose to highlight this risk. If the case did not progress quickly, we did not include the recurrent symptoms in the case but addressed this potential risk during the debrief.*
