# Supplementary material for: Comprehensive 3-Year Pediatric Resident Resuscitation Simulation Curriculum on the Care of the Severely Ill Pediatric Patient
Source: MedEdPORTAL. 2026 Nov 6;22:11650. doi: 10.15766/mep_2374-8265.11650 (PMC13741050; doi:10.15766/mep_2374-8265.11650)
Supplement: Supplementary file 1 [file mep_2374-8265.11650-s001.zip › B. Debriefing Guide.docx]

Appendix B: Debriefing guide

*Instructions: The debriefing guide should be shared with facilitators ahead of the event. It includes an outline of the structure of the educational session as well as discussion points during the debrief to highlight.*

**Structure:**

The pediatric resident resuscitation simulation is a ~3 hour workshop that is aimed at teaching residents about the assessment and management of critically ill children as well as code team leadership skills and team dynamics. The workshop includes three scenarios. For each scenario, the residents will run through a case. Following the case, the facilitators will lead a debriefing discussing the clinical performance as well as team function. Following the debrief, each scenario has an intended procedure or skill that the facilitator will teach and then allow all residents to practice.

There will typically be 4 facilitators and 6-8 residents at each session. They will divide into 2 groups, each with 2 facilitators and 3-4 residents per group. Each group will remain together for 3 cases and will complete the cases in the following order.

**Suggested timeline:**

0:00-0:15 Pretest, introduction and prebrief

0:15-0:25 case 1

0:25-0:45 debrief case 1

0:45-1:00 procedure/skill review for case 1

1:05-1:15 case 2

1:15-1:35 debrief case 2

1:35-1:50 procedure/skill review for case 2

1:55-2:05 case 3

2:05-2:25 debrief case 3

2:25-2:40 procedure/skill review for case 3

2:40-3:00 close/posttest

Cases:

- Year 1
  1. Shock: Distributive/Septic (procedure=intravenous and intraosseous access)
  2. Arrhythmia: Supraventricular tachycardia (procedure=defibrillator use)
  3. Respiratory failure/bradycardia (procedure=bag mask ventilation and neonatal intubation)
- Year 2
  4. Shock: Hypovolemic (procedure=intravenous and intraosseous access)
  5. Arrhythmia: Ventricular fibrillation (procedure=defibrillator use)
  6. Respiratory failure/seizures (procedure=bag mask ventilation and neonatal intubation)
- Year 3
  7. Shock: Obstructive/coarctation (procedure=intravenous and intraosseous access)
  8. Arrhythmia: Asystole (procedure=defibrillator use)
  9. Respiratory failure/hypoventilation (procedure=bag mask ventilation and neonatal intubation)

Procedures:

1. Vascular access (intravenous and intraosseous access)
2. Defibrillator use
3. Airway management (bag mask ventilation and neonatal intubation)

Year 1 Debriefing Guide

Return to introduction page

Case 1: Shock-Distributive/Septic

1. Organization/communication
   a. Did leader assume leadership role? Did all the team members have a defined role?
   b. How well did the learners work as a team?
   c. How was team communication? Use of knowledge sharing, “closed-loop” communication?
2. Assessment/diagnosis of shock including
   a. Differential diagnosis of shock (hypovolemic, cardiogenic, distributive, obstructive)
   b. Differentiating compensated vs hypotensive shock (hypotension late finding)
   c. Recognition of distributive (warm) shock (warm extremities, bounding pulses, flash cap refill, wide pulse pressures, decreased diastolic BP) vs non-distributive (cold) shock (cool, prolonged cap refill, faint pulses, normal/increased diastolic BP, narrow pulse pressure), recognition of tachycardia being early sign/hypotension being late finding
   d. Initial lab evaluation (POC dexi, iSTAT, CBC, CMP, culture, iCa, lactate)
3. Management of septic shock (PALS septic shock algorithm below)
   a. Establishing access (IV vs IO)
   b. Aggressive fluid management during first hour with frequent reassessment (review types of fluids and methods of administering normal saline or lactates ringer via push pull)
   c. Early empiric antibiotics (ceftriaxone and vancomycin within 60m)
   d. Treatment of fluid-refractory septic shock with vasoactive agents (distributive/warm: norepinephrine vs non-distributive/cold: epinephrine)
   e. Recognition and management of hypoglycemia
      i. Rule of 50: 5mL/kg of D10, 10mL/kg of D5

Case 2: Arrhythmia-Supraventricular tachycardia

1. Organization/communication
   a. Did leader assume leadership role? Did all the team members have a defined role
   b. How well did the learners work as a team?
   c. How was team communication? Use of knowledge sharing, “closed-loop” communication?
2. Assessment/diagnosis of arrhythmia
   a. Distinguish supraventricular tachycardia from sinus tachycardia (infants HR >220, children >180, absent/abnormal P wave, typically narrow complex QRS)
   b. Describe how the clinical presentation can differ in infant (irritability, poor feeding, rapid breathing) vs older child (palpitations, SOB, chest pain, lightheaded/fainting)
3. Manage supraventricular tachycardia with pulses and poor perfusion
   a. Identify and demonstrate various types of vagal maneuvers (ice to face, valsalva, straw, carotid massage in older children, not ocular pressure)
   b. Medication (adenosine 0.1mg/kg using rapid bolus technique, max 6mg 1^st^ dose, 12mg 2^nd^ dose)
   c. Cardioversion (0.5-1 J/kg initially, can increase to 2 J/kg)

Case 3: Respiratory failure/bradycardia

1. Organization/communication
   a. Did leader assume leadership role? Did all the team members have a defined role?
   b. How well did the learners work as a team?
   c. How was team communication? Use of knowledge sharing, “closed-loop” communication?
2. Assessment/diagnosis of bradycardia with poor perfusion from respiratory failure
   a. Symptomatic vs asymptomatic bradycardia (hypotension, altered mental status, shock)
   b. Causes of bradycardia
      i. H’s & T’s mnemonic to recall reversible causes including
         1. Hs: Hypovolemia, hypoxia, hydrogen ion (acidosis), hypo/hyperkalemia, hypothermia
         2. Ts: Tension pneumothorax, cardiac tamponade, toxins, thrombosis (pulmonary and coronary)
   c. Upper and lower range of heart rate for concern (<60 danger zone)
3. Management of bradycardia with poor perfusion from respiratory failure
   a. First ensuring proper oxygenation/ventilation to assess if resolves bradycardia
   b. CPR proper technique
      i. Compressions: 100-120 compressions/min, at least 1/3 chest, allow complete recoil, minimize interruptions, use of backboard/stool
      ii. Ventilation: 15:2 if no advanced airway, asynchronous once advanced airway
   c. Timing/dosing of epinephrine
      i. Every 3-5 minutes
      ii. IV/IO
      iii. 0.01mg/kg	0.1ml/kg of 1:10,000 for cardiac arrest (vs 0.01mg/kg of 1,000 for anaphylaxis)

Year 2 Debriefing Guide

Return to introduction page

Case 4: Shock-Hypovolemic

1. Organization/communication
   a. Did leader assume leadership role? Did all the team members have a defined role?
   b. How well did the learners work as a team?
   c. Was there “closed-loop” communication?
2. Assessment/diagnosis of shock including
   a. Differential diagnosis of shock (hypovolemic, cardiogenic, distributive, obstructive)
   b. Differentiating compensated vs hypotensive shock (tachycardia early, hypotension late finding)
   c. Initial lab evaluation (BMP, blood gas, lactate, glucose, calcium, cultures, CBC)
3. Management of hypovolemic shock
   a. Establishing access (IV vs IO)
   b. Aggressive fluid management with frequent reassessment (review types of fluids and methods of administering, push pull bolus)
   c. Recognition and management of hypoglycemia
      i. Rule of 50: 5mL/kg of D10, 10mL/kg of D5

Case 5: Arrhythmia-Ventricular fibrillation

1. Organization/communication
   a. Did the admitting resident assume the role of code team leader? Did they remain in that role throughout event?
   b. Did all the team members have a defined role?
   c. How well did the team verbalize what they were doing?
   d. Was there “closed-loop” communication?
2. Assessment/diagnosis of arrhythmia
   a. Differentiate shockable (SVT/pulseless VT/VF) vs non-shockable (PEA/asystole) rhythms
   b. Differential diagnosis of cardiac arrest
      i. H’s & T’s mnemonic to recall reversible causes including
         1. Hs: Hypovolemia, hypoxia, hydrogen ion (acidosis), hypo/hyperkalemia, hypothermia
         2. Ts: Tension pneumothorax, cardiac tamponade, toxins, thrombosis (pulmonary and coronary)
      ii. Commotio cordis: Ventricular fibrillation and sudden death triggered by a blunt, non-penetrating blow to the chest without damage to the ribs, sternum, or heart
3. Management of cardiac arrest from ventricular fibrillation
   a. CPR proper technique
      i. Compressions: 100-120 compressions/min, at least 1/3 chest, allow complete recoil, minimize interruptions, use of backboard/stool
      ii. Ventilation: 15:2 if no advanced airway, asynchronous once advanced airway
b. Shockable (SVT, VT, VF) vs non-shockable (asystole, PEA) rhythms
   i. Defibrillation/asynchronous mode, initial dose 2J/kg, 2^nd^ 4J/kg, subsequent >/=4J/kg, max 10
   ii. (Vs synchronized cardioversion for SVT 0.5-1J/Kg)
c. Timing/dosing of epinephrine
   i. Every 3-5 minutes
   ii. IV/IO
   iii. 0.01mg/kg	0.1ml/kg of 1:10,000 for cardiac arrest (vs 0.01mg/kg of 1,000 for anaphylaxis)

Case 6: Respiratory failure/Seizure

1. Organization/communication
   a. Was there a leader? Did all the team members have a defined role?
   b. How well did the team verbalize what they were doing?
   c. Was there “closed-loop” communication?
2. Assessment/diagnosis of seizure and subsequent respiratory failure including
   a. Differential diagnosis of seizure (metabolic--hypoglycemia, hyponatremia, hypocalcemia, etc; CNS infection, trauma, toxin/ingestion, hypoxia/hypercarbia, intracranial lesion, fever/infection, stroke, seizure disorder)
   b. Initial lab evaluation (stat glucose, BMP, Mg, ionized calcium, cultures, CBC)
   c. Assessment of respiratory failure secondary to benzodiazepine-induced apnea
3. Management of seizure and subsequent respiratory failure
   a. Midazolam IN 0.2 mg/kg (max 10 mg)
   b. LORazepam IV 0.1 mg/kg (max 4 mg)
   c. Management of respiratory failure due to benzodiazepine-induced apnea
      i. Bag mask ventilation, consideration of intubation based on timeline and resources
      ii. Don’t use a reversal agent, just supportive care with BMV or intubation if necessary

Year 3 Debriefing Guide:

Return to introduction page

Case 7: Shock-Obstructive/Coarctation

1. Organization/communication
   a. Did leader assume leadership role? Did all the team members have a defined role?
   b. How well did the learners work as a team?
   c. Was there “closed-loop” communication?
2. Assessment/diagnosis of shock including
   a. Differential diagnosis of shock (hypovolemic, cardiogenic, distributive, obstructive)
   b. Recognition of ductal dependent lesion including coarctation of aorta
      i. Poor feeding, dyspnea, poor weight gain, shock in first few weeks as ductus arteriosus closes
      ii. Disparity of pulses and BP in arms/legs, systolic ejection murmur along LSB at intercostal space 3-4
3. Management of shock from ductal dependent lesion
   a. Establishing access (IV vs IO)
   b. Fluid management (resuscitation indicated given poor PO/UOP but should be cautious given suspected cardiac anomaly with close monitoring/frequent reassessing)
   c. Medications (Prostaglandins for suspected ductal dependent lesion [recognize risk of apnea, fevers with prostaglandins], inotropic support, early involvement of cardiac specialists)
   d. Recognition and management of hypoglycemia
      i. Rule of 50: 5mL/kg of D10, 10mL/kg of D5

Case 8: Arrhythmia-Asystole

1. Organization/communication
   a. Was there a leader? Did all the team members have a defined role?
   b. How well did the team verbalize what they were doing?
   c. Was there “closed-loop” communication?
2. Assessment/diagnosis of arrhythmia
   a. Differential diagnosis of asystole
3. Management of asystole
   a. CPR proper technique
      i. Compressions: 100-120 compressions/min, at least 1/3 chest, allow complete recoil, minimize interruptions, use of backboard/stool
      ii. Ventilation: 15:2 if no advanced airway, asynchronous once advanced airway
   b. Timing/dosing of epinephrine
      i. Every 3-5 minutes
      ii. IV/IO
      iii. 0.01mg/kg	0.1ml/kg of 1:10,000 for cardiac arrest (vs 0.01mg/kg of 1,000 for anaphylaxis)
   c. Shockable (SVT, VT, VF) vs non-shockable (asystole, PEA) rhythms

Case 9: Respiratory failure/Hypoventilation

1. Organization/communication
   a. Was there a leader? Did all the team members have a defined role?
   b. How well did the team verbalize what they were doing?
   c. Was there “closed-loop” communication?
2. Assessment/diagnosis of respiratory failure including
   a. Differential diagnosis of hypoventilation: brain/brain stem injury, intentional/unintentional/iatrogenic drug ingestion
3. Management of hypoventilation from medications
   a. Bag mask ventilation to support respiratory status
   b. Use of reversal agents (indication for use vs waiting/supporting through symptoms)
      i. Naloxone for narcotic overdose (fentanyl), full (0.1mg/kg) vs partial reverse (1-5mcg/kg), shorter half-life than most narcotics so need to redose
      ii. Flumazenil for benzodiazepine overdose, 0.01mg/kg, max 0.2mg, available but rarely used
      iii. Avoid in cases of chronic use, seizure management. Risk of recurrence of symptoms that led to indication for medication being reversed

Procedure Skills

Return to introduction page

Airway management

1. Bag Mask Ventilation:

- Equipment:
  - Face mask: Smallest that extends from bridge of nose to cleft of chin, covering nose and mouth, not eyes
  - Ventilation bag:
    - Self-inflating: Can be used with or without oxygen source. Cannot use to provide continuous oxygen or positive pressure.
    - Flow inflating/anesthesia bag: Requires oxygen flow to function, typically used by trained/experiences providers.
  - Other: pulse ox, oxygen source, suction, NG/OG tube
- Procedure
  - Place patient in “sniffing” position provided no c‐spine immobilization needed
  - Flex neck forward at level of shoulder while extending head. Avoid hyperextension.
    - >2 years, may need padding under occiput	<2 years, may need under shoulder/upper torso
  - Obtain an adequate seal between face and mask using the “EC” hand position
    - 3^rd^, 4^th^, 5^th^ fingers (form E) positioned along jaw to lift. Thumb/index (form C) make seal, hold mask to face
    - Avoid pressure on soft tissue in submental area which can compress airway
  - Give breath over 1 second, watch for chest rise. Use just enough force to make chest rise. If CPR without advanced airway, ratio of 15:2. If advanced airway, 12-20 breaths per minute.
  - Assess efficacy: Chest rise, improved clinically/saturations, color change on CO2 monitor, audible breath sounds

2. Intubation

- Equipment:
  - SOAP ME: suction, O2, airway, positioning, meds/monitors, EtCO2/other equipment
  - Tube:
    - Size (age/4 +4) (down ½ if cuffed)
    - Cuffed vs uncuffed
  - Blades
    - Miller: straight, infants/younger kids, lift epiglottis directly
    - Mac: curved, older kids/adults, place in vallecula
    - 00=very preterm (<28w), 0=preterm, 1=term/infant, 2= “switch to 2 after 2 years old”, 3=middle school age
- Procedure
  - Open mouth
  - Hold laryngoscope in the left hand and insert blade to the right of tongue, advancing the blade into the hypopharynx, pushing tongue to left
  - Lift the laryngoscope upward and forward, avoid rocking back on teeth
  - Visualize vocal cords. Ask for cricoid pressure as needed
  - Have assistant provide tube to right hand and pass through cords. Insert to ~3 x the tube size.
  - Verify position (CO2 detection, breath sounds, clinical improvement, CXR)
  - Secure tube

Access:

1. IV access

- Equipment:
  - IV start kit, 22-24 gauge IV catheter, primed t-connector with normal saline filled syringe attached, blue clave, comfort measures, arm board, gloves, bandage, tape
- Procedure:
  - Start ¼ to ½ inch distal from point of where bevel should enter vein, hold catheter flat and linear to the vein with the bevel up.
  - While holding skin taut to anchor the vein, insert needle quickly and decisively
  - After initial puncture, stop as most movement occurs after this
  - Advance catheter slowly until flash of blood is seen and/or “pop” felt.
  - With positive blood flashback, advance catheter sheath off needle and into vein
  - Release tourniquet and connect pre-flushed t-connector with normal saline syringe and blue clave to the catheter hub
  - Slowly flush catheter with normal saline observing for infiltration, erythema, or patient complaint of pain. Discontinue IV if symptoms are observed.
  - Secure IV and place clear adhesive dressing over device, leaving insertion site visible.

2. IO access
   - Equipment: EZ-IO driver, needle (various sizes, 5mm line should be visible above skin when inserted to point it is touching the bone. If not, needle is too small)
   - Indications: IO are used if there is a need for rapid vascular access when IV access cannot be obtained. An IO can be used to administer medications given via a peripheral IV such as fluids, blood products, and medications.
   - Contraindications/relative contraindications: fracture/crush injury near site, fragile bones (OI), prior attempt in same bone within 24h, infection in overlying skin
   - Procedure:
     - Sites: proximal tibia preferred, distal tibia, proximal humerus (recommended if >8), distal humerus
     - Insertion: Insertion site is flat part on tibia 1-3cm below/medial to bony prominence. Stabilize leg but don’t put hand behind insertion site. Push needle through skin until you feel bone. Drill until feel decrease in resistance. Remove stylet, attach syringe and aspirate. Infuse.
     - Removal: Generally <24h. Attach a 5-10ml syringe to act as handle. While maintaining axial alignment, twist the syringe and catheter clockwise while pulling straight out. Do not rock or bend. Apply gentle pressure as needed

Defibrillator

- Defibrillation/synchronized cardioversion using ZOLL defibrillator:
  - Attach electrodes to the machine and to patient as noted on pictures on pads
    - For patients less than 8 years old/25 kg, use ZOLL pediatric defibrillation electrodes.
  - Turn selector switch to the red “DEFIB” section
  - Select the energy using up/down arrows
    - Default for adults 120🡪150🡪200J	Default for peds 50🡪70🡪85J
    - Round up as needed
  - If shockable rhythm
    - Hit charge
    - When red button illuminates, clear patient/push shock
  - For synchronized cardioversion
    - Select defib and push the synch on/off button
  - CPR feedback
    - Place electrode with CPR sensor on sternum in location of hands for CPR as indicated on photo on pad
    - Perform CPR normally. Machine will provide feedback on rate, depth, and release

For adult pads only, if rate and depth are appropriate, the diamond will fill with color (PPI, perfusion performance indicator). If recoil time appropriate, the depth bar will fill. Visual and audio prompts will not if too slow or shallow.

For adult and pediatric pads, if rate too slow, rate will light up and a 100 BPM metronome will be heard until correct
