# Supplementary material for: Comprehensive 3-Year Pediatric Resident Resuscitation Simulation Curriculum on the Care of the Severely Ill Pediatric Patient
Source: MedEdPORTAL. 2026 Nov 6;22:11650. doi: 10.15766/mep_2374-8265.11650 (PMC13741050; doi:10.15766/mep_2374-8265.11650)
Supplement: Supplementary file 1 [file mep_2374-8265.11650-s001.zip › C. Equipment.docx]

Appendix C: Equipment

*Instructions: This document outlines the equipment that should be available during the simulation cases and procedural practice. Can be adapted based on local resources to align with materials that would be available to participants in their clinical environment.*

Cases:

- Infant or child manikin based on age of patient in case
- Monitor to display vitals
- Nasal canula
- Oxymask
- Code cart
- Backboard
- Stool (for CPR)
- PALS card
- Airway
  - Self-inflating bag
  - Flow-inflating bag
  - Multiple size masks
  - Multiple size endotracheal tubes
  - Multiple size mac and miller blades
  - Stylet
- Access
  - Bone to insert intraosseous needle
  - IO drill
  - Multiple size IO needles
  - T connector
- Push pull set up
  - IV tubing
  - 3 way stop cock
  - 10 cc syringe
  - IV fluid bag
- Defibrillation
  - Defibrillator
  - Defibrillator pads
- Bag and ice to perform vagal maneuver
- 3 way stop cock for adenosine administration

Procedure

- Airway (1 set up per learner)
  - Airway head
  - Self-inflating bag
  - Flow-inflating bag
  - Multiple size masks
  - Multiple size endotracheal tubes
  - Multiple size mac and miller blades
  - Stylets
- Access (1 set up per learner)
  - IV pad
  - Flush
  - T connector
  - 22 gauge catheters
  - Tourniquet
  - Bone to insert intraosseous needle
  - IO drill
  - Multiple size IO needles
  - T connector
- Defibrillation
  - Defibrillator
  - Defibrillator pads
