# Supplementary material for: Comprehensive 3-Year Pediatric Resident Resuscitation Simulation Curriculum on the Care of the Severely Ill Pediatric Patient
Source: MedEdPORTAL. 2026 Nov 6;22:11650. doi: 10.15766/mep_2374-8265.11650 (PMC13741050; doi:10.15766/mep_2374-8265.11650)
Supplement: Supplementary file 1 [file mep_2374-8265.11650-s001.zip › E. Simulation Prebrief Sample Template.docx]

Appendix E: Simulation Prebrief Sample Template

*Instructions: This is a sample script that can be used during the prebrief prior to starting the simulation to orient the learners and support psychological stafety for the upcoming debriefs*.

1. Basic Assumption^1^

*“We believe that everyone participating in these simulation activities is intelligent, capable, cares about doing their best and wants to improve.”*

2. Fiction Contract

*“I have done everything I can to make this as real as possible, but in the end, it is not reality; I have to depend on you. I ask you to do your best to act as if this is real so that the time we have together is used to our best advantage. Act like you would in the situation I will present. We will have time to answer questions and discuss the case following the scenario.”*

3. Goals/Objectives/Confidentiality

*“Today’s session is going to focus on your teamwork in taking care of a critically ill patient. Regardless of if you persue primary care, hospital medicine, or a fellowship for your future career, it is important that you can provide the initial management to severely ill patients. This is meant to be an opportunity to learn. You are not being tested or evaluated. It is ok to make mistakes. What happens here stays here.”*

4. Orientation to roles/environment

- Timeline/format: “*We will run through three cases. First, we will do the case, then we will spend time discussing how things went and any questions you have. After the debrief, we will practice some related procedures. The session will take about three hours total.”*

- Who am I? Who are you? Where are we?: *“You will be assuming the role of residents in each of the cases. For each scenario we will let you know where the case is taking place (such as the emergency department of an acute care patient room. We will let you know who else is in the room and how to contact additional team members.”*

- Review equipment/resources: “*We will now review the equipment in the room and the manikin. I will first let you listen to the manikin in their normal healthy mode so you can learn what the features are. If you ever have questions about what the manikin is supposed to be showing or want findings that the manikin is not able to tell you like cap refill, verbalize that you are assessing those features and I will give you the findings.”*

1. Center for Medical Simulation Basic assumption. Center for Medical Simulation website. Published 2024. Accessed April 16, 2026 https://harvardmedsim.org/resources/the-basic-assumption/
