# Supplementary material for: Comprehensive 3-Year Pediatric Resident Resuscitation Simulation Curriculum on the Care of the Severely Ill Pediatric Patient
Source: MedEdPORTAL. 2026 Nov 6;22:11650. doi: 10.15766/mep_2374-8265.11650 (PMC13741050; doi:10.15766/mep_2374-8265.11650)
Supplement: Supplementary file 1 [file mep_2374-8265.11650-s001.zip › F. Pre- and Postsession Learner Survey.docx]

Appendix F: Pre/post survey

*Instructions: This should be provided to leaders prior to and immediately following the educational activity.*

| Presurvey  Please rate how much you agree with the following statement: | | | | | |
| --- | --- | --- | --- | --- | --- |
| I feel confident in my ability to *recognize:* | | | | | |
|  | Strongly Agree | Somewhat Agree | Neither Agree nor Disagree | Somewhat Disagree | Strongly Disagree |
| Shock |  |  |  |  |  |
| Cardiac arrhythmia |  |  |  |  |  |
| Respiratory failure |  |  |  |  |  |
| I feel confident in my ability to *manage:* | | | | | |
|  | Strongly Agree | Somewhat Agree | Neither Agree nor Disagree | Somewhat Disagree | Strongly Disagree |
| Shock |  |  |  |  |  |
| Cardiac arrhythmia |  |  |  |  |  |
| Respiratory failure |  |  |  |  |  |
| I feel well prepared to *perform* the following skills without supervision: | | | | | |
|  | Strongly Agree | Somewhat Agree | Neither Agree nor Disagree | Somewhat Disagree | Strongly Disagree |
| Airway management |  |  |  |  |  |
| Vascular access |  |  |  |  |  |
| Defibrillation |  |  |  |  |  |

| Postsurvey  Please rate how much you agree with the following statement: | | | | | |
| --- | --- | --- | --- | --- | --- |
| I feel confident in my ability to *recognize* | | | | | |
|  | Strongly Agree | Somewhat Agree | Neither Agree nor Disagree | Somewhat Disagree | Strongly Disagree |
| Shock |  |  |  |  |  |
| Cardiac arrhythmia |  |  |  |  |  |
| Respiratory failure |  |  |  |  |  |
| I feel confident in my ability to *manage* | | | | | |
|  | Strongly Agree | Somewhat Agree | Neither Agree nor Disagree | Somewhat Disagree | Strongly Disagree |
| Shock |  |  |  |  |  |
| Cardiac arrhythmia |  |  |  |  |  |
| Respiratory failure |  |  |  |  |  |
| I feel well prepared to *perform* the following skills without supervision: | | | | | |
|  | Strongly Agree | Somewhat Agree | Neither Agree nor Disagree | Somewhat Disagree | Strongly Disagree |
| Airway management |  |  |  |  |  |
| Intravascular Access |  |  |  |  |  |
| Defibrillation |  |  |  |  |  |
|  | Strongly Agree | Somewhat Agree | Neither Agree nor Disagree | Somewhat Disagree | Strongly Disagree |
| The simulation developed my clinical reasoning skills |  |  |  |  |  |
| The simulation helped me to recognize patient deterioration early |  |  |  |  |  |
| I received feedback during the debriefing that helped me to learn |  |  |  |  |  |
| This was a valuable learning experience |  |  |  |  |  |
| Free Text Questions: | | | | | |
| What, if anything, is one thing you learned during the session? |  | | | | |
| Please provide any additional feedback about this workshop and how it could be improved. |  | | | | |

| Facilitator Feedback: | | | | | |
| --- | --- | --- | --- | --- | --- |
|  | Strongly Agree | Somewhat Agree | Neither Agree nor Disagree | Somewhat Disagree | Strongly Disagree |
| The facilitator showed competence and knowledge in the subject area being covered. |  |  |  |  |  |
| The facilitator was an effective teacher. |  |  |  |  |  |
| The facilitator’s discussions were clear and organized. |  |  |  |  |  |
| The facilitator stimulated interest in the subject. |  |  |  |  |  |
| The facilitator made me feel comfortable and at ease during the debriefing |  |  |  |  |  |
| Please provide any additional feedback for the facilitator |  | | | | |
