# Supplementary material for: Comprehensive 3-Year Pediatric Resident Resuscitation Simulation Curriculum on the Care of the Severely Ill Pediatric Patient
Source: MedEdPORTAL. 2026 Nov 6;22:11650. doi: 10.15766/mep_2374-8265.11650 (PMC13741050; doi:10.15766/mep_2374-8265.11650)
Supplement: Supplementary file 1 [file mep_2374-8265.11650-s001.zip › G. Facilitator Observation Form.docx]

Appendix G: Facilitator observation form

*Instructions: This document is for the facilitators to use while observing the cases to take notes on observations to provide content for debrief.*

| What went well | Area for improvement |
| --- | --- |
| Questions brought up to address | Any significant concerns to be sure to address |
